# Metabolomics in the wild: A scoping review of non-invasive methods for understanding vertebrate wildlife health and success

**DOI:** 10.1371/journal.pone.0359720

**Published:** 2026-10-26

**Authors:** Rebecca Mogey, Royston Goodacre, Ffion Curtis, Michelle Maden, Katie L. Edwards

**Affiliations:** 1 North of England Zoological Society, Chester Zoo, Upton-by-Chester, United Kingdom; 2 Centre for Metabolomics Research, Department of Biochemistry, Cell and Systems Biology, Institute of Systems, Molecular and Integrative Biology, University of Liverpool, Liverpool, United Kingdom; MARE – Marine and Environmental Sciences Centre, PORTUGAL

## Abstract

An increase in metabolomics research over the last decade has expanded species-specific knowledge of biological processes, but has primarily focused upon humans, domesticated and model animal species. Recent advances, particularly around data management pipelines for untargeted studies, make the field more accessible to disciplines outside those embedded in analytical chemistry, including wildlife conservation. To establish the breadth of existing wild vertebrate animal metabolomic research, specifically utilising non-invasive sampling, this scoping review establishes: (1) general trends in publication numbers; (2) coverage of wildlife taxonomic groups and species; (3) subject numbers and sample size; (4) non-invasive sample types; (5) metabolomic techniques utilised; (6) the context of current literature and (7) conservation implications. This scoping review was conducted following the JBI methodology and reported in accordance with PRISMA-ScR; searches were conducted using PubMed, Scopus, Web of Science and supplemented with Google Scholar; following screening, 83 peer-reviewed original research papers were identified using predefined criteria and data extracted. The most popular areas of research were physiology and reproduction (30%) and diet and nutrition (27%). Although most publications focused on a single species, there were 11 multi-species publications, resulting in metabolomics data for 93 species. A large proportion of which, 72%, were mammalian, and of these, 52% are currently listed at risk of extinction on the IUCN Red List. With over 60% of all publications published between 2022–2025, non-invasive vertebrate wildlife metabolomics appears to be gaining traction as a tool in wildlife physiology research. However, future research would benefit from alignment with model animal metabolome research experimental design, as few studies included the consideration of metabolite extraction optimisation, storage conditions, the use of integrated omics platforms beyond microbiome analysis and consideration of statistical power, particularly in respect to intra-individual variation and the characterisation of species-specific metabolomes.

## Introduction

Metabolomics is the study of small biomolecules (typically <1,500 Da) within cells, biofluids, tissues or whole organisms, involved in metabolic processes. Individually, these small molecules are referred to as metabolites; collectively, they are known as the metabolome [1]. These molecules are substrates and products of metabolism and are therefore influenced by both genetic (intrinsic) and environmental (extrinsic) exposures [2]. Metabolomics or metabolite profiling therefore represents a read out of the molecular phenotype of the system under study and is routinely used in both human and other animal biomedical research for biomarker discovery, with many well-characterized markers robustly predicting relevant clinical outcomes across a variety of treatments and populations [3]. Recent advances in the field of metabolomics, particularly around data management pipelines for untargeted studies, are making the field more accessible to disciplines outside those embedded in analytical chemistry [4].

Several reviews have been published in relation to mammalian metabolomics. Many of these assess the vast amount of published work relating to metabolomic changes in animal models of human disease, which expand the knowledge of the physiopathology of various human illnesses, such as depression [5], cancer biology [6], metabolic disease [7] and reproductive pathology [8]. In addition, many studies and subsequent reviews have been completed for companion animal research; e.g., for the domestic dog, with several canine diseases showing some promise for potential biomarker and/or disease mechanism discovery, highlighting the growth of metabolomics in veterinary research over the last decade [9]. Metabolomics analysis in many domesticated animals, such as agricultural livestock species, has been extensively reviewed. Findings highlight that the majority of studies have previously focused on biofluid analysis in various fields of bovine research, including animal physiology, with a number of important species and sample types, including non-invasive faecal material, under-represented by comparison [10]. More recently, a review has discussed findings from nearly 100 studies which assess metabolomic changes associated with pathologies, such as mastitis and respiratory disease in dairy cows [11–13].

The expansion of research in domesticated animal metabolomics over the last decade is enabling farmers, veterinarians and researchers to, for example, ascertain disease propensity [14], assess fertility [15] and analyse milk quality [16]. The Livestock Metabolome Database (LMDB) now provides over 1,200 metabolite entries (LMDB - https://lmdb.ca/ assessed on Oct 2026), allowing researchers to conduct more targeted metabolomics studies. However, this database is currently limited to captive bovine, ovine, caprine, equine and porcine species and by comparison, characterisation of the metabolome in wild animal species is suspected to be extremely limited – without similar efforts resulting in a centralized database.

A review completed in 2012 highlighted that metabolomic research in animal wildlife has great potential for both clinical and nutritional research in exotic animals [17]. Although a recent review completed on marine organisms has again described metabolomics as an indispensable tool, it is thought that metabolomics research in the context of vertebrate wild animal species could be lagging far behind their domesticated counterparts [18]. Sampling, particularly in free-ranging wild animals, presents a huge obstacle. Although sampling from captive animal wildlife species (such as a zoo-housed animal) can be considered, often sampling remains limited to those types which are non-invasive, as capture and subsequent handling not only presents challenges from a licencing and ethical standpoint, but represents a potential source of stress for the animal, possibly changing physiological parameters measured [19]. However, sampling conducted in conjunction with routine veterinary procedures, as well as voluntary participation sampling, where appropriate, are extremely valuable and have been used to gain greater understanding of the biology and health related changes in several species, such as the Sumatran rhinoceros (*Dicerorhinus sumatrensis*) [20]. It is also important to note that storage and transport of samples remains challenging in non-clinical settings.

According to a review published in *Science* in 2016, at the turn of the century we entered a period defined by the substantial impacts of humans on the Earth’s biogeography [21], with current extinction rates up to a thousand times higher than they would be if people were not in the picture, largely due to habitat loss and climate change [22]. Indeed, climate change is currently the biggest security threat this planet has ever faced. Metabolomics represents a potentially untapped resource of non-invasive chemical markers of animal physiology, health and wellbeing, which could be used as a tool to improve conservation efforts for endangered species. However, the knowledge, expertise and financing required to utilise these techniques appropriately are perhaps not yet widely available or utilised effectively within the wildlife conservation sector [23].

To establish the breadth of existing wild vertebrate animal metabolomic research utilising non-invasive sampling, this scoping review will aim to review: (1) general trends in publication numbers; (2) coverage of wildlife taxonomic groups and species; (3) subject numbers and sample size; (4) non-invasive sample types; (5) metabolomic techniques utilised; (6) the context of current literature and (7) if their metabolomics studies are directly linked to conservation efforts by the authors. To our knowledge, this is the first scoping review to examine the application of metabolomics in non-invasive wildlife research. We are conducting this review to gain vital insight into how metabolomics may be contributing to the health, well-being and ultimately the conservation of wild vertebrate animal species.

## Methods

A scoping review was chosen to provide an overview of the existing literature, establishing the breadth of wild vertebrate animal metabolomic research, utilising non-invasive sampling. The proposed scoping review was conducted in accordance with the *Journal of Biological Enquiries* (*JBI*) methodology for scoping reviews [24]. The PRISMA-ScR checklist was used to ensure completeness [25] (S1 Checklist). A protocol was created in advance of the review based on the input and expertise of the co-authors, which include metabolomics (RG), wildlife physiology (KLE) and health data science (MM/FC). The final protocol was registered with the open science framework: https://archive.org/details/osf-registrations-gntp9-v1

### Inclusion criteria

This review was confined to ‘vertebrate animal wildlife’, ‘metabolomics’ and ‘non-invasive sampling’.

Studies were included where all criteria were met as described in detail below:

- Population: Animal species were included as ‘vertebrate animal wildlife’ animal species were included if they (a) are not considered to be domesticated (subjected to long-term intentional selective breeding by humans from a wild or natural state to life in close association and to the benefit of humans) and (b) are not considered to be an animal model (a non-human species used for the purposes of conducting (or improvement of) of biomedical research). Genetically, wild animals maintain the diversity inherent within their species, evolving through natural selection, whereas domesticated animals generally are not good proxies for their wild counterparts due to significant differences in behaviour, genetics and physiology, meaning these animals are metabolically dissimilar from wild animals. Conversely, even when bred in captivity, wild animal species, such as the Asian elephant *(Elephas maximus)* have not been domesticated. Compared with canine and feline pets for example, domesticated ~5000 years ago, breeding of wild animals has been happening for relatively few generations, and although it is accepted that some inadvertent selection may occur in captive breeding programmes, there is an absence of long-term intentional selective breeding by humans [26–28].
- Concept: Metabolomics is defined as the qualitative and/or (semi) quantitative measurement and analysis of small molecule metabolites using targeted or untargeted analytical approaches, including but not limited to liquid chromatography coupled with mass spectrometry (LC-MS), gas chromatography coupled with MS (GC-MS), nuclear magnetic resonance (NMR) spectroscopy, Fourier transform infrared (FTIR) or Raman spectroscopy and imaging MS. The measurement of hormone metabolites only, known as wildlife endocrinology, has been extensively reviewed and is not included in this scoping review unless accompanied by other analyte measurement.
- Context: This scoping review has only considered publications that have utilised sample types collected using non-invasive (no manipulation or penetration of the body) and minimally invasive collection methods (for example, gentle squeezing or catheterisation under anaesthetic). Studies where animals were euthanised as part of the research conducted were not considered to be non-invasive and thus not included.
  In the UK, research using animals is governed by the Animals (Scientific Procedures) Act 1986 (ASPA). Under this Act, a study that involves applying procedures on living vertebrates for scientific purposes are required to be covered by Home Office Licencing (unless performed for recognised veterinary, agricultural or animal husbandry practice). A procedure is regulated “if it is carried out on a ‘protected animal’ for a scientific or educational purpose and may cause that animal a level of pain, suffering, distress or lasting harm equivalent to, or higher than, that caused by inserting a hypodermic needle according to good veterinary practice” and must be individually assessed to adhere to the principles or guidelines covered by ASPA. Although recognised as a methodological limitation, blood samples were excluded from this review as both the circumstances and purpose of blood collection were not consistently reported across studies in preliminary searches and unfortunately resource did not allow exploration into localised authorisation and/or appropriate licensing to ensure adherence to our ‘minimally invasive’ criteria. Although three cases of urinary catheterisation were included, despite these being conducted under veterinary anaesthesia, these could be manually checked under the scope of this review for ethics and licensing approvals and allowed all identified publications where urine, a frequent non-invasive sampling technique particularly in zoological settings, to be included.
- Date range: 2011–2025. Methodology in metabolomics has advanced rapidly, particularly over the last decade, to remain as relevant to current methodologies and research strategies as possible this review only included articles published from 2011 onwards.

### Search strategy

The following three databases were searched, Scopus, PubMed and Web of Science. All three databases were searched from 2011 to Dec 31^st^, 2025. Database searches were supplemented with a Google Scholar search with the first 200 titles screened for inclusion [29]. An example of a full search strategy for Scopus is presented in Supplementary Material S2 File.

### Study selection and data charting

All identified references from searched databases were exported, collated and uploaded into Rayyan software for screening of sources (https://www.rayyan.ai/) and any duplicates removed. A two-part study selection process was then utilised. First, the title and abstract were screened by the primary reviewer (RM) against inclusion criteria; second, the full text of articles not excluded based on title or abstract review were then retrieved and assessed by the primary reviewer. At both stages, > 10% of the articles were independently reviewed by two secondary reviewers (KLE and RG) against the same criteria. Any disagreements where recorded and resolved between the three reviewers.

### Data extraction and synthesis

Data extraction and collation was performed in Microsoft Office Excel (https://www.microsoft.com/en-gb/microsoft-365/excel) by the primary reviewer. Data were presented as text, tables and where deemed appropriate, graphically for visual interpretation to create a narrative structured around the included publication breadth: (1) general trends in publication numbers; (2) coverage of wildlife taxonomic groups and species; (3) subject numbers and sample size; (4) non-invasive sample types; (5) metabolomic techniques utilised (6) the overall research aim and (7) reference to conservation of populations. Subject number and sample size were sought from mammalian papers only due to large taxonomic and sample type biases making comparisons across taxa inappropriate and grouped for simplification. Primary research aims were broadly categorised into five groups to discuss breadth and trends in current literature aims.

An assessment of quality beyond reference to sample and subject number was not performed as part of this scoping review as we felt that recommendations for future research regarding the design and reporting would not be appropriate given the variety of study approaches taken by publications included in the review. Moreover, when assessing endangered animals, it is perhaps not surprising that sample numbers are low, and this limitation in sample sizes is highlighted where appropriate.

## Results

Once searches were completed, duplicates were removed leaving 461 articles identified for title and abstract review; following this review, 367 articles were excluded and 94 articles included for full text review (Fig 1). Reasons for exclusion after full text review (*n* = 11) included, incorrect population where domesticated or laboratory model animals were the subject (*n* = 3), incorrect methodology where metabolomics was not employed as a primary or secondary methodological approach (*n* = 1), incorrect sampling where an invasive sampling technique was used, or samples were obtained from animals euthanised in conjunction with the research conducted (*n* = 4) or finally, where the article was relevant but not published in English (*n* = 3). The flow diagram (Fig 1) shows article identification and inclusion criteria.

**Fig 1:**
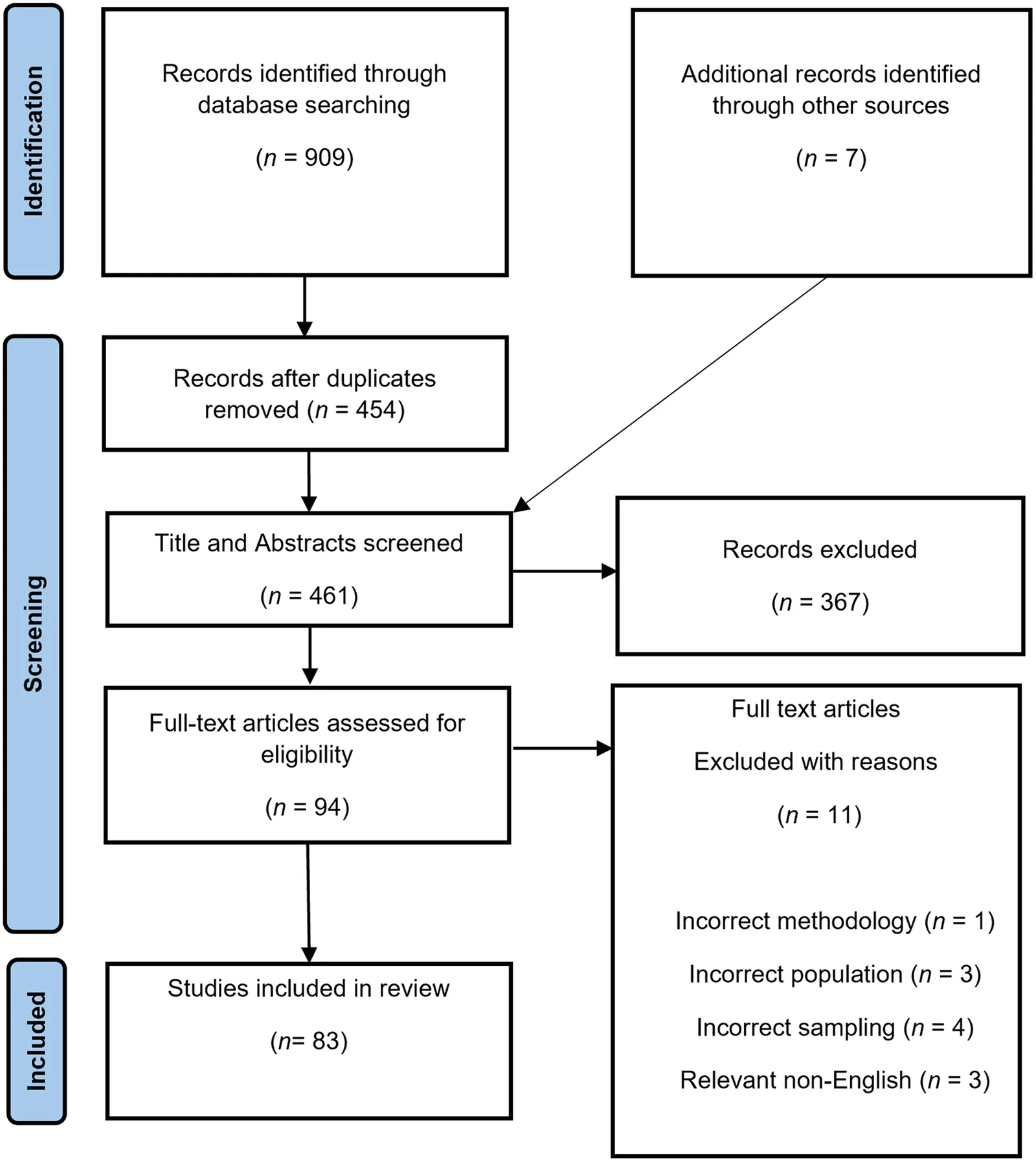
PRISMA 2020 diagram of the search and article selection process. The flow diagram illustrates the number of references identified, screened, assessed for eligibility and included in this scoping review of metabolomics in wild animal vertebrate species, as well as reasons for exclusion at full text review.

### Characteristics of included articles

A total of 83 articles met the inclusion criteria and were included in this scoping review, after a consensus was reached by all authors. These included 82 original research articles and one review, from which one publication was found to comply with the acceptance criteria of this review. These 83 publications were included (Table 1) and reviewed as below.

**Table 1:** Characteristics of included publications. This table includes a summary of all included articles (publications) in this review of metabolomics in wild animal vertebrate species using non-invasive methodology (*n* = 83).

Species	Class^a^	IUCN Status^b^	Matrix^c^	Analytical technique^d^	Research Aim	Study summary	Ref.
*Acinonyx jubatus*	M	VU	F(NI), U (MI)	LC-UV-Vis	Physiology and reproduction	Investigating the absorption, metabolism and excretion of dietary isoflavones in cheetahs. Absorbed isoflavones were efficiently conjugated, primarily as sulfate metabolites but only a small proportion was absorbed. Lower sulfation capacity than in domestic cats may increase the potential biological activity of isoflavones	[30]
*Acinonyx jubatus*	M	VU	U (NI)	GC-MS	Health and disease	Untargeted metabolomic study to document urine organic acids in healthy cheetahs. Findings included specific organic acids related to the dysregulation of coenzyme A metabolism which correlate with age. This study provides urine organic acid reference values for captive cheetah, providing a foundation for future research	[31]
*Acinonyx jubatus*	M	VU	U (MI)	LC-MS, GC-MS	Physiology and reproduction	To generate new hypotheses for future pathophysiological mechanisms of chronic disease, both serum and urine amino acid profiles of healthy cheetah were examined. No amino acid concentrations differed significantly between males and females, but some were significantly affected by age. This study provides baseline information on serum and urine amino acid profiles of healthy cheetah	[32]
*Acinonyx jubatus*	M	VU	F (NI)	LC-MS	Health and disease	Metabolomic profiling provides insight into confounding factors that are not found in care records. Here the detection of an antibiotic in a non-treated animal, likely transferred through grooming, showed that metabolomic analysis can enhance omics studies by enabling the correct grouping of this animal for analysis.	[33]
*Acinonyx jubatus*	M	VU	U (MI), S (UD)	NMR	Diet and nutrition	Captive cheetahs are often subject to diets that are low in glycine abundant collagen-rich animal fibre, production of which may be limited in several species. Metabolomic analysis of urine and serum showed that glycine supplementation influenced several metabolomic pathways, although the implications of these findings on cheetah health are unknown.	[34]
*Ailuropoda melanoleuca*	M	VU	F (NI)	GC-MS	Analytical techniques	The aim of this research was to establish an optimized extraction protocol for the analysis of adult and juvenile giant panda faecal metabolome. Results demonstrated that that two of six extraction solvents were the most appropriate to be used in future protocols to gain insight into the gut host-microbiome relationship.	[35]
*Ailuropoda melanoleuca*	M	VU	U (NI)	LC-MS	Physiology and reproduction	Pregnancy determination in the giant panda is challenging, this study explored pregnancy-related changes of urinary metabolites when compared with anoestrus (determined using endocrinology analysis), identifying differential metabolites. This provides new insights into metabolomic changes in the giant panda, providing a basis for determining pregnancy.	[36]
*Ailuropoda melanoleuca*	M	VU	F (NI), U (NI), Sa (NI), S (UD)	NMR	Physiology and reproduction	Characterisation and quantification of the metabolome of healthy giant panda across four biological matrices (faecal, urine, serum and saliva). Faecal and urine sex and age molecular profile differences were described. This work provides a framework, allowing the acceleration of further -omics research	[37]
*Ailuropoda melanoleuca*	M	VU	F (NI), S (UD)	LC-MS	Diet and nutrition	Little is known about giant panda faecal and serum metabolites. The untargeted analysis of both matrices found a significant correlation between the gut microbiota and faecal metabolites and sex and age differences in serum metabolites only, suggesting separate feeding should be considered in relation to age and sex	[38]
*Ailuropoda melanoleuca*	M	VU	U (NI)	LC-MS	Physiology and reproduction	Urinary metabolomics and microbiomics were used to assess if physiological stress could be contributing to decreased natural mating and thus poor population growth of captive giant panda. Results connect altered urinary microbe composition and arginine and neurotransmitter synthesis to poor libido and could be used to improve reproductive success	[39]
*Ailuropoda melanoleuca*	M	VU	H (NI)	GC-MS	Analytical techniques	Hair metabolomics has been used in environmental toxicological studies; therefore, the giant panda fur metabolome could reflect environmental exposure. Eight extraction protocols were evaluated, differing extraction solvents were found to be optimal for amino acid/secondary metabolites and fatty acid extraction, providing extraction protocols for future study	[40]
*Ailuropoda melanoleuca*	M	VU	U (NI)	LC-MS	Physiology and reproduction	Mate choice is important in natural mating success in captive giant pandas. Urinary metabolome analysis shows loss of natural mating behaviour could be linked to tryptophan and arginine metabolic pathways and the decrease of androgens. Findings indicate changes in stress-related metabolic pathways caused by frustration may be responsible for natural mating decline	[41]
*Ailuropoda melanoleuca*	M	VU	F (NI)	GC-MS	Physiology and reproduction	Sex pheromone signals are important in solitary animals such as the giant panda; this study explored which gut microbes and microbial metabolites are involved in the synthesis of giant panda sex pheromones. Findings indicate that volatile metabolites such as faecal acetate are involved in mate preference, enriching our understanding of sex pheromone synthesis	[42]
*Ailuropoda melanoleuca*	M	VU	F (NI)	LC-MS	Diet and nutrition	Age-linked changes of giant panda metabolome and microbiome could be used to understand how giant pandas adapt to their herbivorous diet. Results show that transitioning from a milk-dominant to a bamboo-specific diet impact both metabolome and microbiome and that the gut microbiota-bile acid axis is key in age-related metabolism with new insight into lipid metabolism	[43]
*Ailuropoda melanoleuca*	M	VU	F (NI)	LC-MS	Diet and nutrition	Understanding foraging behaviour is important, as changes in climate can impact food availability for wild giant panda. Metabolomic and microbiome results show higher faecal sugar content and altered microbiome related to fibre on consuming bamboo stem, suggesting that habitat enrichment with bamboo is important to promote natural foraging strategy	[44]
*Ailuropoda melanoleuca*	M	VU	F (NI)	LC-MS, GC-MS	Physiology and reproduction	Scent marking is a way in which giant pandas communicate. Field scent marking sites and faecal material metabolome, and microbe community were analysed. Microbial species composition is likely responsible for specific scent signalling compounds at marking sites, providing insight into chemical communication with faecal material not sharing the same function as scent marking	[45]
*Ailuropoda melanoleuca*	M	VU	U (NI)	LC-MS	Physiology and reproduction	Reduced reproductive capacity in giant panda may be due to the stress of incompatible pairings in captivity. Urinary metabolomics indicated that dysfunction of amino acid metabolic pathways could be implicated, together with urine cortisol correlation with atypical mating behaviour demonstrating that physiological stress induced by incompatible pairings may impact natural breeding behaviours	[46]
*Ailuropoda melanoleuca*	M	VU	U (NI)	LC-MS	Physiology and reproduction	This study aimed to investigate urinary metabolomics of different oestrous stages in giant panda. Results showed that metabolites varied significantly throughout the differing stages of oestrus, some metabolites correlated with oestrogen level fluctuation while differing metabolites fluctuated with progesterone levels, highlighting potential metabolites for oestrus monitoring for breeding management	[47]
*Ailuropoda melanoleuca*	M	VU	U (NI)	LC-MS	Analytical techniques	Immunoassay techniques for monitoring steroids are hindered by species-specific metabolism; this study presents a validated steroidomics method for detecting a wide range of steroids in urine. Several steroids in giant panda urine were revealed, these results advance biomonitoring techniques in breadth and identification	[48]
*Ailuropoda melanoleuca*	M	VU	U (NI)	LC–MS	Physiology and reproduction	Although critical for captive breeding, pregnancy monitoring in giant panda is difficult with traditional immunoassays. Urinary metabolomics/ steroidomics revealed several discriminative features which when used as a biomarker panel may improve early pregnancy diagnosis, improving pregnancy monitoring and our knowledge of giant panda reproduction	[49]
*Ailuropoda melanoleuca*	M	VU	U (NI), Se (NI) (AG)	LC-MS	Physiology and reproduction	Natural mating is more effective than artificial insemination in the giant panda, with mate choice an important factor in breeding success. This study analysed metabolic changes in mate preference trials during oestrus. Several metabolites and metabolic pathways were potentially associated with female mate preference, providing knowledge for future breeding management	[50]
*Ailuropoda melanoleuca*	M	VU	F (NI)	LC-MS	Diet and nutrition	The diet of the giant panda consists predominantly of bamboo; this unique adaptation is of considerable scientific interest. Feeding different bamboo species is shown to have significant effects on both the metabolome and microbiome, with *P. iridescens* perhaps a substitute for the routinely used *Phyllostachys* species (*P. edulis*) in captive management of giant pandas	[51]
*Ailurus fulgens*	M	E	F (NI)	LC-MS	Diet and nutrition	Understanding the taxonomical and functional differences within the gut microbiota is an important consideration in the red panda, known for its reliance on bamboo. Results indicate the consumption of bamboo shoots heighten the presence of pathogenic bacteria and that semi-free environment animals show increased microbial diversity, these findings offer improved captive breeding management	[52]
*Alouatta pigra*	M	EN	F (NI)	GC-MS	Diet and nutrition	Shifts in the gut microbiome and microbial metabolism in response to seasonal nutritional availability remain uncharacterised in black howler monkeys. Strong relationships were found between specific diet plant metabolites and the faecal metabolome and seasonal diet changes and gut microbiome function, suggesting the gut microbiome buffers against variations in nutrient intake	[53]
*Alytes obstetricans*	A	LC	Se (NI) (Skin)	LC-MS	Health and disease	The pathogenic fungi *Batrachochytriumdendrobatidis* causes chytridiomycosis, which threatens amphibian populations. Although the skin microbiome can provide some protection, its overall functionality lacks understanding in midwife toads. Skin mucosal metabolomics and microbiome analysis revealed that *Bd* infection alters microbiome taxonomic and functional profiles, predicts *Bd* disease and identifies future biomarkers of infection	[54]
*Anas platyrhynchos*	B	LC	F (NI)	NMR	Diet and nutrition	Changes in habitat or food supply can cause physiological stress and subsequent process activation to restore homeostasis in waterfowl. Metabolomic analysis during changes in feed availability found that metabolite profiles could determine feed restricted mallard ducks. Faecal metabolomics shows promise as a tool for monitoring the stress response in captive wild bird species	[55]
*Capra ibex*	M	LC	F (NI)	LC-MS, GC-MS	Diet and nutrition	Little is known about the structure and function of the faecal microbiome or metabolome of wild ruminants. Samples revealed seasonal changes in composition and functional profiles in Alpine ibex and confirmed phenotypic plasticity of the host-associated microbiome, necessary to buffer dietary changes. Findings provide insight and support the holobiont framework views	[56]
*Carollia perspicillata*	M	LC	F (NI)	GC-MS	Diet and nutrition	Fruit secondary metabolites can have a role in the defence of fruit pulp against frugivores such as the fruit bat, but frugivore mutualistic interactions remain unexplored. The ingestion of secondary metabolites deterred bat foraging behaviour and altered the absorption of nutrients and the faecal metabolome. This furthers our knowledge of fruit chemistry on fruit-eating animal physiology	[57]
*Cervus albirostris*	M	VU	F (NI)	LC-MS	Ecology and evolution	The evolution of unique nutrient metabolism in harsh environments is poorly understood. Here the gut microbiota and metabolome of the white-lipped deer were characterised across differing environments. Extreme winter environmental condition significantly altered the composition, function, and metabolism of gut microbiota. This work highlights gut microbiota as key in environmental resilience in wild, high-altitude mammals	[58]
*Cervus elaphus kansuensis*	M	LC	F (NI)	LC-MS	Diet and nutrition	The composition and function of the gut microbiota in Gansu red deer is yet uncharacterised. The composition of gut microbiota in captive and wild red deer was shown to be significantly different, with the most differential metabolites involved in lipid metabolism. This study shows differential diets and living environments have led to gut microbiome adaptations in wild *vs.* captive animals	[59]
*Chrysocyon brachyurus*	M	NT	U (NI)	GC-MS	Physiology and reproduction	It is suspected that urinary chemical signals secreted by male maned wolves induce female ovulation. This study assessed urinary volatile organic compounds and found differing compounds between intact males and immature males or females, several of which are implicated in other mammal reproductive communication studies and has identified candidates for further behavioural bioassay study	[60]
*Crocodylus porosus*	R	LC	F (NI)	LC-MS	Health and disease	Optimisation of welfare is essential for farmed saltwater crocodiles. Utilising multi-omics analyses, this study examined the stress response of farmed saltwater crocodiles in differing housing conditions. Although corticosterone levels (immunoassay) showed no significant difference, grouped crocodiles showed metabolic disruption. Results demonstrate importance of the faecal metabolome and microbiome in animal welfare assessments	[61]
*Cryptobranchus alleganiensis alleganiensis*	A	VU	Se (NI) (Skin)	LC-UV-Vis	Health and disease	The pathogenic fungi *Batrachochytrium dendrobatidis* (*Bd*) has been detected on eastern hellbenders but infection rates are lower than in other species, possibly due to antifungal metabolites present. Metabolite fingerprinting of wild animal skin secretions revealed Bd detection was not associated with metabolite composition or richness, providing insights into amphibian health and disease mechanisms	[62]
*Dicerorhinus sumatrensis, Diceros bicornis, Ceratotherium simum, Rhinoceros unicornis*	M	CE, CE, NT, VU	F (NI)	NMR	Health and disease	Iron overload disorder has been identified in many wildlife species, including the rhinoceros, with some species more susceptible than others. Comparison of the faecal microbiome and metabolome of four rhinoceros species showed more susceptible species had decreased microbial diversity. This work shows that gut microbial community is implicated in iron acquisition	[63]
*Eira barbara, Lontra longicaudis, Galictis vittata, Galictis cuja*	M	LC, NT, EN, LC	F (NI)	LC-MS	Ecology and evolution	Non-invasive sampling has great potential in the study of wild animal species. Here a metabolomic approach was used to characterise the metabolites present in Neotropical mustelid faecal material. Chemical profiles allowed species-specific identification and species showed high metabolome diversity. This research supplements non-invasive methods in the study of mustelids	[64]
*Eptesicus fuscus*	M	LC	F (NI)	LC-MS	Ecology and evolution	Vespertilionid bats are exceptionally long lived when compared to similar sized mammals and at present bat faecal metabolic profile alterations with age have not been explored. Metabolites associated with tryptophan metabolism and protein digestion were found to differentiate young *vs.* elderly brown bats. Findings provide further insight into the physiological mechanisms of bat longevity	[65]
*Giraffa camelopardalis*	M	VU	U (NI)	NMR	Physiology and reproduction	Giraffe urinary metabolome is unexplored; anaesthetic related complications make immobilization of giraffe risky practice. Molecules quantified give information in relation to diet, digestion and gut microbial co-metabolism allowing the effects of age and sex to be assessed. Provides preliminary information on the urinary metabolome and could trigger non-invasive method development for health monitoring in giraffe	[66]
*Giraffa camelopardalis*	M	VU	F (NI)	GC-MS	Physiology and reproduction	The faecal metabolome of the giraffe has thus far not been characterised. Distinct variations in metabolome profiles were observed in relation to external stimuli such as season, supplementary feeding and sex. This work assists the development of non-invasive techniques for the assessment of health and ultimately conservation of wildlife species such as the giraffe	[67]
*Giraffa camelopardalis, Ceratotherium simum, Damaliscus pygargus*	M	VU, NT, LC	M (MI)*	NMR	Physiology and reproduction	Differences in milk metabolome composition and metabolomic pathways in wild taxa is relatively unexplored. Here, the milk metabolome in several species was assessed and identified that the metabolome and several metabolic pathways were specialised within species. Limitations included comparison with other research reports on related species and further work is required to draw species-specific conclusions	[68]
*Gorilla gorilla gorilla*	M	CE	F (NI)	GC-MS	Ecology and evolution	Gut microbes are key in the understanding of mammalian biology as they heavily influence host physiology. Faecal microbial communities and gut metabolomes of wild lowland gorilla groups were characterised. Group differences appeared to relate to geographical range feeding behaviour and additionally, anthropogenic pressure. This work provides an alternate view on the evolution of the gut microbiome of wild primates	[69]
*Gorilla gorilla gorilla, Gorilla beringei beringei*	M	CR, CR	F (NI)	GC-MS	Ecology and evolution	Little is known about the evolution of primate gut microbiomes. The faecal microbial communities and metabolomic profiles in lowland and mountain gorillas were characterised and shown to have distinct patterns. However, these differences become less apparent during similar dietary constraints. This study suggests that diet is implicated in the species-specific microbiome patterns currently observed in primates.	[70]
*Lagopus muta* (ssp*. japonica*)	B	LC (not listed)	F (NI)	LC-MS	Diet and nutrition	Feeding management is a potential reason for the suboptimal survivability of reintroduced captive-raised Japanese rock ptarmigans. Analysis of faecal metabolites and gut microbiome showed strong differences between *in situ* and *ex situ* individuals. This reveals important work is required for the development of the gut microbiome when considering future species reintroduction	[71]
*Afrixalus paradorsalis, Arthroleptis sylvaticus, Arthroleptis poecilonotus, Didynamipus sjostedti, Hyperolius ocellatus, Leptopelis modestus, Phlyctimantis boulengeri, Phrynobatrachus auritus, Xenopus fraseri*	A	LC, LC, LC, VU, LC, LC, LC, LC, DD	Se (NI) (Skin)	LC-MS	Analytical techniques	Understanding the composition of small organic molecules found on the skin of amphibians may reveal selective resistance to the pathogenic fungus *Batrachochytrium dendrobatidis*. This study has developed and assessed a non-invasive foam-tipped swab technique allowing the characterisation of small molecules found on the skin of amphibians. Findings reveal significant correlation between chemical profile and species	[72]
*Leontocebus weddelli*	M	LC	F (NI)	GC-MS	Ecology and evolution	Little is known of the gut microbiome and its mechanisms and therefore dietary requirements of small bodied wild primates. Analysis of the microbiome and metabolome provides insights into the metabolome and microbiome with potentially environmentally derived dominant bacterial taxa enabling saddleback tamarin dependence on digestible carbohydrates found in fruits and plants	[73]
*Leptonychotes weddelli, Mirounga leonina*	M	LC, LC	F (NI)	NMR	Ecology and evolution	There is limited knowledge of the gut microbiome for species living in remote locations. Comparison of faecal microbiota and metabolomic profiles of Antarctic seal species revealed that microbial communities were significantly different but metabolites similar. Results indicate that functional redundancy is present in gut microbiota of seals and advances our understanding of Antarctic animal coevolution between host and the gut microbiota	[74]
*Lophophorus lhuysii*	B	VU	F (NI)	LC-MS	Diet and nutrition	The Chinese monal is endangered with limited captive populations and further insight into biological mechanisms required to expand populations. Analysis of the gut microbiome and metabolome between wild and captive animals for nutritional status and digestion information showed significant differences. This study provides foundational information for future research in this species	[75]
*Macaca mulatta*	M	LC	F (NI)	LC-MS	Health and disease	Although the gut microbiota has been linked with the stress response, the molecular pathways linking physiological stress and gut microbiota are unclear. In this study,stress significantly altered rhesus macaque faecal microbial composition and function. This work suggests that modifications of the microbiota could mitigate stress related health declines, making it focal for wildlife health monitoring	[76]
*Macaca mulatta*	M	LC	F (NI)	LC-MS	Ecology and evolution	The adaptation of rhesus macaques to high altitude environments has been heavily researched but the role of the gut microbiota in this adaptation remains unclear. This study suggests the gut microbiota plays not only a role in adaptation to high altitude but that this adaptation also aids degradation of exogenous toxins	[77]
*Macaca mulatta*	M	LC	F (NI)	LC-MS	Ecology and evolution	Animal adaptations for survival in extreme environmental conditions such as high altitude may be associated with gut microbial composition. Faecal metabolome and microbiome analysis revealed metabolites and microbes linked to Bilirubin (implicated in red blood cell turnover) were enriched in high altitude animals. Environmental stress caused by living at high altitude therefore affects microbiota composition	[78]
*Macropus rufus*	M	LC	F (NI)	GC-MS	Health and disease	In captivity, red kangaroos often suffer from various diseases, which may be linked with the gut microbiota. This study investigated the impact of the probiotic *Clostridium butyricum* on both the faecal microbiota and metabolites and showed that supplementation effects on metabolism may be independent of microbiota composition and suggests that the probiotic alters metabolism	[79]
*Manis pentadactyla, Manis javanica*	M	CR, CR	F (NI)	LC-MS	Diet and nutrition	Captive pangolins face many challenges, including intestinal health issues. This study compared the gut microbiota and metabolome of pangolin species and identified significant differences in the makeup and variety of microbiota and corresponding metabolome of differing species. This study provides important data for the improvement of captive pangolin feeding and health management	[80]
*Moschus berezovskii*	M	EN	H (NI)	LC-MS	Health and disease	Captive endangered forest musk deer are prone to diseases which effect overall animal health. The comparison of hair metabolomics between healthy and diseased deer found several differential metabolites and functional pathways correlating with disease. Hair metabolomics has significant potential as a non-invasive diagnostic tool in the detection of disease in forest musk deer	[81]
*Mus musculus*	M	LC	U (NI)	GC-MS	Physiology and reproduction	Mouse urine contains a variety of molecules that serve as olfactory signals. Metabolomics and proteomics were used to identify key components of chemical signalling. This study identified that composition of urinary metabolites and proteins differ between sex, are heavily influenced by environment and there is a strong selection on systems of sexual signalling during speciation	[82]
*Mus musculus*	M	LC	Se (V) (NI)	GC-MS	Physiology and reproduction	Saliva and vaginal fluids are sources of chemical signals in mammal species and thus may act as signals of health and reproductive status. Analysis revealed changes in vaginal (but not salivary), microbiome composition during the reproductive cycle of wild mice and high correlation between microbiome, proteome and metabolome providing strong evidence of interaction	[83]
*Neophocaena asiaeorientalis*	M	EN	F (MI)*	LC-MS	Physiology and reproduction	The gut microbiota is integral for both foetal and maternal health during pregnancy of the captive Yangtze finless porpoise. Both microbial community and metabolome during reproductive stages show significant alterations. This study provides insight into faecal microbiota and metabolome changes which could help effective reproductive management of the species	[84]
*Neophocaena asiaeorientalis*	M	EN	U (NI)	LC-MS	Physiology and reproduction	Successful captive breeding of the critically endangered Yangtze finless porpoise is important to maintain vital populations. To gain further insight into reproductive biological processes, the urinary metabolome was characterised during pregnancy. This study provides valuable urinary metabolome information for management during pregnancy and potential diagnostic markers of pregnancy	[85]
*Neophocaena asiaeorientalis*	M	EN	F (MI)*	LC-MS	Diet and nutrition	It is known that the gut microbiota can act as a buffer when nutritional availability is restricted. Here seasonal dietary variation is seen to impact both the gut microbiome and faecal metabolome in the Yangtze finless porpoise. The plasticity of the microbial community within this species and correlation with metabolome could be used to aid conservation initiatives	[86]
*Neophocaena asiaeorientalis*	M	CE	F (MI)*	LC-MS	Health and disease	Captive Yangtze finless porpoise are subjected to social separation which can cause physiological and psychological stress. Analysis of the gut microbiome and metabolome during mother-calf separation found increased microbial taxa associated with disease and changes in the metabolome in mother-separated individuals. This study sheds new light on the negative effects of captive endangered mammal social separation	[87]
*Neophocaena asiaeorientalis*	M	CE	F (MI)*	LC-MS	Physiology and reproduction	Seasonal breeding pattern knowledge of the critically endangered Yangtze finless porpoise is critical for conservation efforts. Analysis of the faecal microbiome and metabolome showed significant compositional and metabolic pathway alterations between breeding and non-breeding seasons, providing new insights into how seasonal reproductive behaviour correlates with gut microbes and metabolites, both in captivity and in the wild	[88]
*Neotoma bryanti Neotoma lepida*	M	LC	F (NI)	LC-MS, NMR	Ecology and evolution	Little is known about how mammals respond to shifts in resources, this study began to characterise the foraging ecology of two species of wood rat through metabolomic and gut microbiota analysis, allowing the detailed examination of habitat diet specific metabolic processing. This work provides insight into nutritional ecology from which future work can be built upon	[89]
*Orcinus orca*	M	DD	B (NI)	LC-MS, GC-MS	Health and disease	Climate change, as well as other anthropogenic stressors are significantly changing ocean ecosystems, so methods to monitor the health of marine life are vital. This study showed the collection and analysis of exhaled breath condensate and provide the assessment of metabolomic profile variation in healthy killer whales. This work describes the potential of breath analysis in marine mammals	[90]
*Palaeornis eupatria*	B	LC	F (NI)	LC-MS	Diet and nutrition	Current gut microbiome and metabolome knowledge in the critically endangered Alexandrine parakeet is limited. Analysis of the gut microbiome and metabolome showed that dietary changes altered the concentration of several gut metabolites, metabolic pathways and thus faecal metabolites. The relevance of this data to improvement of health in the species requires further exploration	[91]
*Panthera pardus (*ssp*. delacouri*)	M	VU (CR)	F (NI)	LC-MS	Diet and nutrition	Gut microbes are essential for environmental adaptation of wild animals to their environment but have been seldom researched in the wild North China leopard. Distinct differences in the faecal metabolic profiles of wild and captive animals were found, representing the first insight into microbiota composition and the metabolome for this species	[92]
*Panthera tigris*	M	E	F (NI)	LC-MS	Health and disease	Irritable bowel syndrome is a disease suffered by captive south China tigers. The faecal microbiome and metabolome of healthy *vs.* animals with mild diarrhoea were compared. It was found that mild diarrhoea stool had significantly lower microbial diversity than healthy stool and suggested that adding a daily probiotic to diets could improve species gut health	[93]
*Parus major*	B	LC	F (NI)	LC-MS	Physiology and reproduction	The microbiome of birds is known to be associated with disease risk, but knowledge of underlying mechanisms is limited. The faecal metabolome of wild great tits was analysed. No correlation was found with respect to age, sex and body condition but season was shown to significantly alter the metabolome, suggesting there is an environmental impact on metabolomic composition of great tits	[94]
*Propithecus coquereli*	M	CE	F (NI)	NMR	Diet and nutrition	Studies investigating the consequences of foliage quality on animal gut microbiomes are lacking. This work examined the pace of gut microbiome structure and function shift during dietary changes in sifaka and found more diverse foliage promoted higher microbiome diversity. This study highlights the vulnerabilities of specialist folivore gut microbiomes for future management of populations	[95]
*Pseudacris crucifer*	R	LC	Se (skin) (NI)	LC-MS	Ecology and evolution	Few studies have considered how contaminants in the environment affect the microbiome. Acute exposure to fly ash did not significantly affect bacterial communities or metabolite profiles in adult spring peepers, suggesting relative resistance to brief contaminant exposure. However, laboratory housed frogs altered bacterial community structure. Research highlights the need for further research on the effects of environmental contaminants	[96]
*Rangifer tarandus*	M	VU	F (NI)	LC-MS	Diet and nutrition	Reindeer must gain body fat throughout summer to cope with harsh environments. This study demonstrated that captive and wild reindeer gut microbial composition, functions and metabolic profiles are significantly different, highlighting captivity as a gut microbial modulator in reindeer providing new insights into feeding and management practices that could support conservation of populations	[97]
*Rhinopithecus brelichi*	M	CE	F (NI)	LC-MS	Health and disease	Deworming treatments are often used to protect the critically endangered gray snub-nosed monkey, but parasite infection and treatment disrupt host gut microbiota. Treated individuals showed deworming significantly increased richness and diversity of gut microbiota and significantly altered the metabolome. The gut microbiota and metabolites could enhance delivery of probiotic and prebiotic treatments, reducing side effects	[98]
*Rhinopithecus roxellana*	M	E	U (NI)	GC-MS	Ecology and evolution	The Sichuan golden monkey has the highest known distribution of the three Chinese snub-nosed monkey species, as such conservation efforts have focused on aspects of ecology and adaptation. In this study urinary metabolomics found a number of metabolites associated with metabolic responses to sex or season. Results show underlying adaptation of these species in response to environmental factors which could aid future conservation strategies	[99]
*Rhinopithecus roxellana*	M	E	F (NI)	LC-MS	Diet and nutrition	How different conservation strategies affect the gut microbiota remains under researched. Assessment in golden snub-nosed monkeys showed that captivity caused distinct microbiota-metabolite co-variation patterns. Results show that naturalistic diets are associated with microbial and metabolic profiles similar to those in free ranging animals. This work highlights the importance of microbiome in future conservation practices	[100]
*Salvelinus namaycush*	Ac	LC	Se (NI) (P)	LC-MS, NMR	Physiology and reproduction	Limited knowledge of underlying molecules has hindered further exploration of sexual preferences for chemical signalling in lake char. Using metabolomics, this study found that male lake char use taurocholic acid, a partial match of juvenile odour, as a mating pheromone. This furthers understanding of pheromone communication may improve population management of both native and invasive populations	[101]
*Silverstoneia flotator*	A	LC	Se (NI) (Skin)	LC-UV-Vis	Health and disease	Some of the most severe outbreaks of *Batrachochytrium dendrobatidis* infection have occurred at high elevation sites. This study found the skin bacterial communities and metabolite profiles of the rainforest rocket frog differed at one lowland site. This study furthers knowledge of skin metabolites in relation to animal health and permitting future exploration of the mechanisms of disease vulnerability	[102]
*Spheniscus demersus*	B	CE	F (NI)	LC-MS	Physiology and reproduction	Successful egg production in the endangered African penguin is important in maintaining breeding populations. This study identified that both gut microbial community and faecal metabolites were significantly correlated with egg production, giving insight into the gut microbiota, metabolome and reproduction and furthering the understanding of mechanisms in abnormal egg production	[103]
*Spheniscus demersus*	B	CE	F (NI)	LC-MS	Physiology and reproduction	Age is associated with increased pathogenic bacteria in the gut of African penguins. Analysis of the gut microbiome and faecal metabolome showed that age is an influencing factor correlating significantly with certain gut microbial species and faecal metabolites. These results provide foundational information on gut microbial composition and faecal metabolites, vital for the management of this species	[104]
*Suricata suricatta*	M	LC	BO (NI)	GC-MS	Analytical techniques	Mammalian olfaction provides information such as identity and sex and is an essential method of communication, but difficulty obtaining high quality samples has hampered research efforts. This study evaluated the use of thermal desorption tubes for non-invasive sampling of meerkat body odour and provided optimised methodology, allowing non-invasive assessment of chemical signals	[105]
*Tapirus bairdii*	M	EN	F (NI)	LC-MS	Diet and nutrition	The use of prebiotics in herbivore species has increased in recent years, but little is known about the metabolic effects. The analysis of metabolites and metabolic pathways in Baird’s tapir following inulin supplementation revealed significant enrichment in comparison with control groups. This work provides data on the effects of prebiotics on gut metabolites, highlighting the need for further investigation	[106]
*Teratoscincus roborowskii*	R	LC	F (NI)	LC-MS	Diet and nutrition	The frog-eyed gecko is frugivorous and has been observed to consume grapes. Evaluation of both the gut microbiota and faecal metabolite composition demonstrated shifts consistent with food type with strong correlation between diet, gut microbiota and faecal metabolites. Findings provide a base knowledge for host health and captive breeding of desert lizards	[107]
*Tursiops truncatus*	M	LC	B (NI)	LC-MS	Health and disease	Health monitoring in common bottlenose dolphins can be difficult as the collection of biological samples is challenging. This study performed metabolomic analysis on exhaled breath and compared this to several measured health parameters in animals with lung abnormalities. Metabolites correlated with typical health measurements, highlighting breath analysis as a tool for the non-invasive assessment of health in wild cetaceans	[108]
*Tursiops truncatus*	M	LC	B (NI)	LC-MS, GC-MS	Analytical techniques	Health monitoring in common bottlenose dolphins can be challenging. This study describes the analysis workflow needed to characterise exhaled breath metabolites and provides the first cetacean exhaled breath library. Breath analysis provides a future wildlife conservation tool, providing a non-invasive method to further our understanding of marine mammal physiology	[109]
*Ursus arctos, Ursus thibetanus*	M	LC, VU	F (NI)	LC-MS	Ecology and evolution	Due to sampling constraints, brown and black bear species on the Qinhai-Xizang Plateau are understudied. Joint analysis of the microbiota and faecal metabolites of both bear species were correlated, and significant metabolic differences were found between species. This study provides insights into the wildlife ecology of wild bears in this unique environment	[110]
*Varecia variegata rubra, Varecia variegata, Mandrillus sphinx, Pan troglodytes, Hylobates lar, Cebus olivaceus, Gorilla gorilla, Ursus thibetanus, Ursus arctos, Nasua nasua, Lycaon pictus, Canis lupus, Panthera tigris, Panthera pardus, Panthera leo, Felis chaus, Felis margarita, Hyaena hyaena, Ceratotherium simum, Equus burchelli, Loxodonta africana, Elephas maximus*	M	CR, CR, VU, EN, EN, LC, CR, VU, LC, LC, EN, LC, EN, VU, VU, LC, LC, NT, NT, NT, EN, EN	F (NI)	LC-MS, GC-MS	Ecology and evolution	It is unknown if the diverse gut microbiota communities found in different animal species are in fact functionally redundant. This study identified that mammalian metabolomes are diverse and correlated to the microbiome, with species-specific enriched metabolites. This demonstrates that differences in gut microbial composition are linked to distinct metabolic profiles and diverse gut microbial communities which perform different functions	[111]
*Propithecus diadema, Indri indri, Avahi laniger, Lepilemur mustelinus*	M	CR, CR, VU, VU	F (NI)	NMR	Ecology and evolution	The gut microbial composition could drive host niche differentiation. This was explored by comparing gut microbiota and metabolites of four Madagascan lemur species with different dietary specialisations. The lemurs had species-specific microbiomes, metagenomes and metabolomes, revealing specialised gut microbiota and gastrointestinal structures which perhaps facilitate differential feeding ecologies	[112]

This table includes extracted characteristics from all included articles (publications) in this review of metabolomics in wild animal vertebrate species using non-invasive methodology (*n* = 83).Abbreviations: ^a^ Animal class: M, mammal; B, bird; A, amphibian; R reptile and Ac: Actinopterygii. ^b^ IUCN status: DD, data deficient; LC, least concern; NT, near threatened; VU, vulnerable; EN endangered or CR, critically endangered (Version 2024−1. https://www.iucnredlist.org). ^c^ Biological matrix: F, faecal; U, urine; B, breath; H, hair; BO, body odour; M, milk; S serum, Sa saliva and Se, secretion (P, pheromone; AG, anogenital gland; V, vaginal) and if they were collected NI, non-invasively or MI, minimally-invasively or UD, Undetermined– not included in this review. *Manual manipulation required for collection ^d^ Analytical technique: LC-UV-Vis, Liquid Chromatography ultraviolet visible spectroscopy; LC-MS, liquid chromatography mass spectrometry; GC-MS, gas chromatography mass spectrometry; NMR, nuclear magnetic resonance spectroscopy.

### General trends

During the earliest years of the review period (2011–2013), this review retrieved only one publication, suggesting a limited awareness or accessibility of metabolomic techniques in non-invasive vertebrate wildlife research until 2014 (Fig 2). A notable peak in publications was seen in 2020, suggesting a growing interest and possibly an improved accessibility to not only metabolomic technologies, but perhaps sampling techniques, as well as accessibility to sampling. It should be noted that although a surge in publication numbers within biomedical fields specifically occurred during the COVID-19 pandemic [113], specialised fields like wildlife metabolomics are unlikely to have been impacted to such a degree. Although speculative, this increase may reflect pandemic-related restrictions that limited laboratory and field work, allowing a focus on manuscript preparation and subsequent publication. Following an observed decrease in 2021, there is an overall increasing trend in publications compared with previous years, with over 60% (52/83) of all publications published between 2022–2025. This perhaps reflects a rapidly emerging research area gaining momentum as a tool for wildlife health and/or conservation monitoring. Despite this increase, this remains extremely low relative to human and vertebrate model animal publications. Although methodological and computational developments have made metabolomics techniques more accessible to all, barriers that may have encumbered this growth could be both the challenges associated with non-invasive sample collection within wild populations, processing requirements for non-invasive sample matrices, and the annotation/identification of metabolites in non-model systems where libraries are more limited.

**Fig 2:**
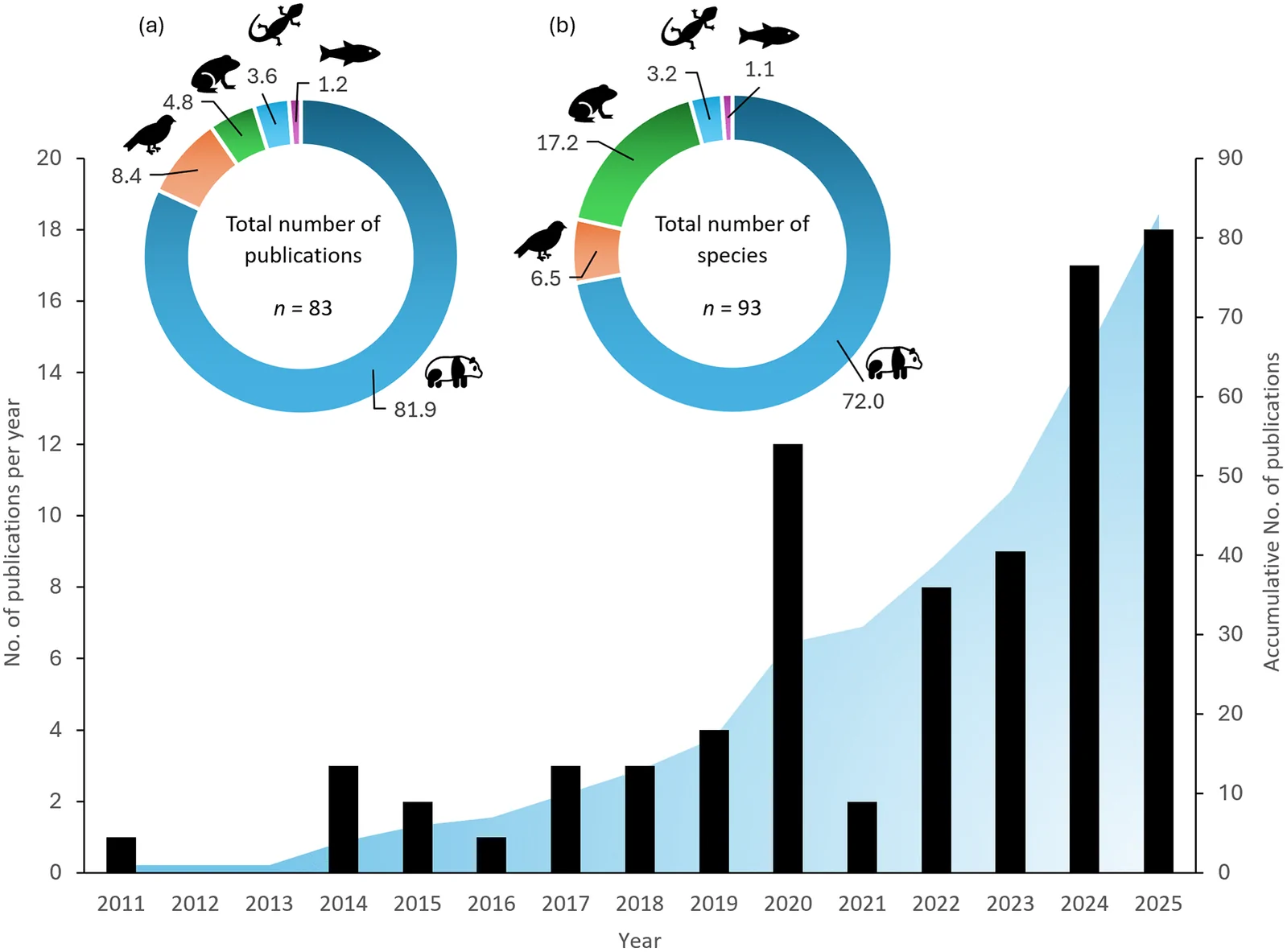
Publication date trends of included articles (publications) and proportion of taxonomic classes represented in the reviewed publications applying non-invasive metabolomics to wildlife vertebrate species. The total number (bars) and accumulative count (shaded blue curve) of non-invasive vertebrate wildlife metabolomic research publications from searches detailed within this review (2011-2025) by year of publication (*n* = 83 peer reviewed publications). The pie charts illustrate the distribution of included (a) publications by taxonomic group (*n* = 83), including Mammalia (dark blue; 81.9%) Amphibia (green; 4.8%), Aves (orange; 8.4%), Reptilia light blue; 3.6%) and Actinopterygii (pink; 1.2%) and (b) unique species by taxonomic group (*n* = 93), including Mammalia (dark blue; 72.0%), Amphibia (green; 17.2%), Aves (orange; 6.5%), Reptilia (light blue; 3.2%) and Actinopterygii (pink; 1.1%).

### Current trends and gaps in coverage of wildlife species and IUCN Red List status

A large proportion of the metabolomic studies identified in this review focused on mammalian species, representing 81.9% (68/83) of the publications retrieved in the primary search. Other vertebrate classes were comparatively underrepresented (Fig 3), including Amphibia (4/83; 4.8%), Aves (7/83; 8.4%), Reptilia (3/83; 3.6%) and Actinopterygii (1/83; 1.2%). The suitability of non-invasive sampling approaches such as the collection of excreta (faeces and urine) from mammalian species for metabolomic analysis could play a part in this mammalian bias; however, the predominance of mammals highlights an already well-established favouritism towards species which are more accessible, well-characterised and of higher public interest, with major conservation journals showing not only a bias towards mammals but specifically to larger, ‘charismatic’ animals like the giant panda *(Ailuropoda melanoleuca)* [114,115]. This uneven taxonomic coverage may constrain the ability of researchers to compare metabolomic studies across a diverse vertebrate range. Several species (*n* = 13) were represented across multiple publications, with the giant panda the most represented species (*n =* 17 publications). Although most publications focused on a single species, there were 11 multi-species publications, making the total number of species across all publications *n* = 93, including Mammalia (67/93; 72.0%), Amphibia (16/93; 17.2%), Aves (6/93; 7.0%), Reptilia (3/93; 3.2%) and Actinopterygii (Ray-finned fishes; 1/93; 1.1%) (Fig 3; S3 File).

**Fig 3:**
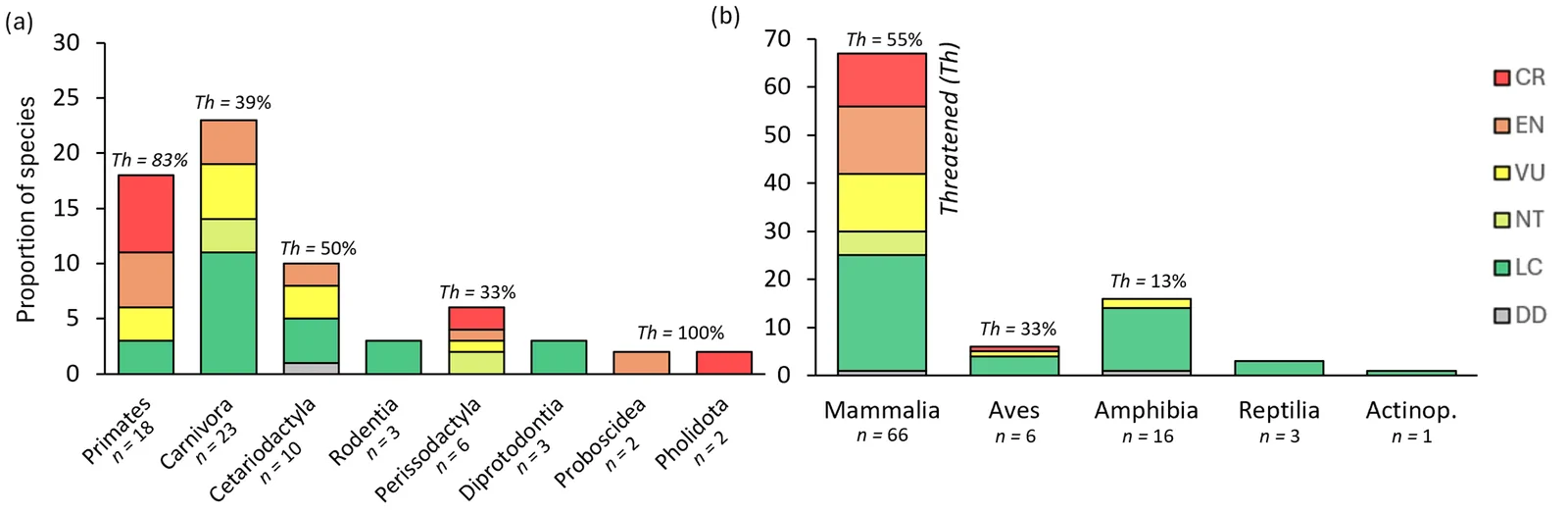
Proportion of taxonomic classes of wildlife vertebrate species where non-invasive metabolomics has been applied, represented by the IUCN Red List. Bar charts illustrate proportion of species (a) across represented Mammalia orders, and (b) across included taxonomic groups. Colours indicate level of threat DD (grey), LC (green), NT (light green), VU (yellow), EN (orange) and CR (red) with threatened (Th) categories (Inclusive of VU, EN and CR) highlighted (%).

Threat status of a species has been previously shown to be weakly associated with presence/absence of research, but the substantial taxonomic biases present in research funding mean that even in well-funded groups such as mammals, capital is often focused on a small selection of species, leaving many threatened species with little or no support [116]. This bias could limit understanding of metabolic responses in rare or declining non-mammalian taxonomic groups. Expanding metabolomic research to encompass a broader range of taxa and conservation statuses would allow the application of metabolomics as a tool for proactive conservation physiology and evidence-based management across vertebrate species.

Of the eight Mammalia orders represented within this review, the proportion of threatened species varied; within Rodentia and Diprotodontia, threatened species were not represented; whereas species within the orders Proboscidea and Pholidota were all threatened. Currently, the IUCN red list reports 26% of assessed mammal species classified as threatened, with reported proportions of threatened species within the orders of Primates (65%) [117,118], Carnivora (27%) [119] and Cetariodactyla (Cetacean 26%) [120] equalling or exceeding this average mammalian threat rate. Comparatively, there is notably a higher proportion of threatened species within these orders included in this review, with 83% of Primates (15/18), 39% of Carnivora (9/23), 50% of Cetariodactyla (5/10) and 67% of Perissodactyla (4/6) categorised as threatened species. This pattern may reflect a research emphasis on conservation-reliant species, driven by the urgent need to understand and preserve populations at risk of extinction. In addition, the term ‘Conservation’ appeared in the main body of text of 66% (55/83) of publications included in this review, highlighting studies motivated by or potentially relevant to conservation, underscoring it as a central theme in non-invasive vertebrate wildlife metabolomics research. However, evidence of applied conservation, where findings were then used or demonstrated in a conservation context, for example as part of a conservation management programme, were not part of this review’s scope.

### Current trends and gaps in mammalian subject numbers and sample size

In the context of this scoping review, the subject/s (individual animal/s) and overall sample size is an important consideration when evaluating potential limitations of current literature. To determine the robustness of study design and thus the extent to which the conclusions can be meaningfully compared across mammalian studies, subject number was divided into five categories, ≤ 5 subjects, > 5 and ≤ 25, > 25 and ≤ 50, > 50 and not declared (ND) – where the number of individuals was unclear or group sampling was used, without individual identification. Almost 60% (40/68) of all mammalian publications had subject groups comprising of ≤ 25 individuals, seven of which comprised of only ≤ 5 individuals (Fig 4). A previous review highlighted that the number of subjects used in livestock research was smaller than those found in publications relating to human or model animal subjects, with approximately half all publications comprising of ≤ 30 subjects [10]. This review surmised that this could reflect limited funding for agricultural research, and this could be the same in the case of wildlife research, compounded by the complexity of potentially limited (often fewer than 25 subjects of the target species are available for sampling) and opportunistic sample collection of wildlife samples.

**Fig 4:**
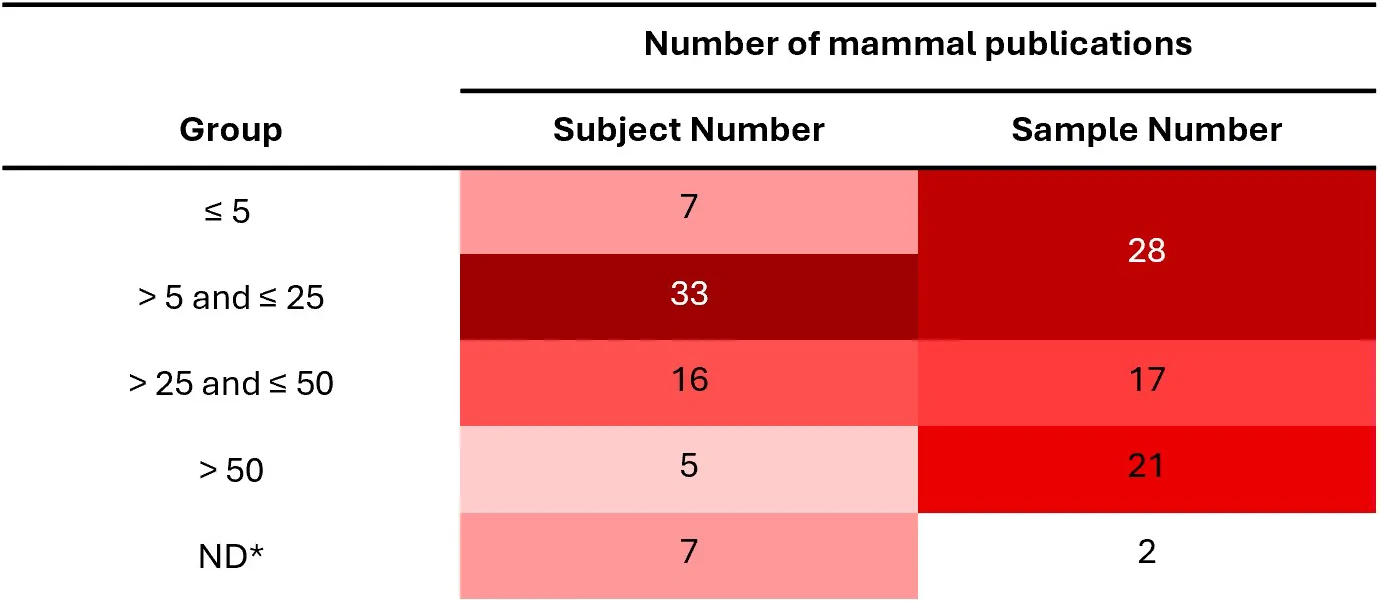
Heatmap displaying the density (number) of publications for both number of individuals and the number of samples in mammalian metabolomics publications. This heatmap shows the group distribution of individual subject and sample number for all mammalian wildlife publications in metabolomics *(n* = 68), with darker colours reflecting larger sample sizes.

Overall, sample size was also relatively small in comparison to model species publications, where hundreds of samples are often included. Sample size was divided into four groups, ≤ 25 samples, > 25 and ≤ 50, > 50 and ND. Over 40% (28/68) of the included mammalian publications had 25 or fewer samples overall. Limited sample sizes are a documented feature of animal metabolomics literature. Although there are no explicit sample size standards for metabolomics studies, largely due to the unknown magnitude of the measured effect, smaller sample sizes generally reduce the statistical power and therefore the robustness of interpretation. This has been highlighted in human studies where the power of the study can be limited depending on the number of individuals analysed [121]. This is particularly pronounced in metabolomic studies, where high dimensionality and cross-validated predictive metrics depend strongly on sample size [122,123]. Although it is important to note that sampling frequency could be influenced by the research question, animal metabolomics studies often highlight small sample size (together with animal species bias) as a limitation of the analyses, perhaps highlighting the need for publications to identify themselves as ‘preliminary’ or ‘pilot’ studies where sample size is limited, with follow up studies then basing sample size selection on this experimental pilot data [124]. The determination of adequate sample size is not only an ethical responsibility but an economical one, with larger cohort sizes significantly escalating study costs, making the assessment of sample size adequacy for animal model studies an important part of study design for scientific, economic and ethical reasons, currently a consideration and justification gap in non-invasive vertebrate wildlife metabolomic research.

Nine of 68 mammalian publications failed to provide either subject number or sample size, omitting a crucial element in scientific reporting. Repeated measures (multiple samples taken from the same individual) improve statistical power by controlling for within individual variation, reducing the risk of overinterpretation [125]; however, 37% (25/68) of mammalian publications, where sample and individual numbers were apparent, only analysed one sample per individual animal and would have benefited from more robust study design. While there is a weakly positive relationship between number of mammalian wildlife species subjects and total number of samples per publication (Fig 5; *R*^2^ = 0.137), variability is evident. Some of the included publications had wildly different sample numbers despite a similar number of animals, indicating that study design across publications typically does not include a similar frequency of collection per individual or indeed a higher frequency sample collection when there are a low number of subjects (e.g., longitudinal studies).

**Fig 5:**
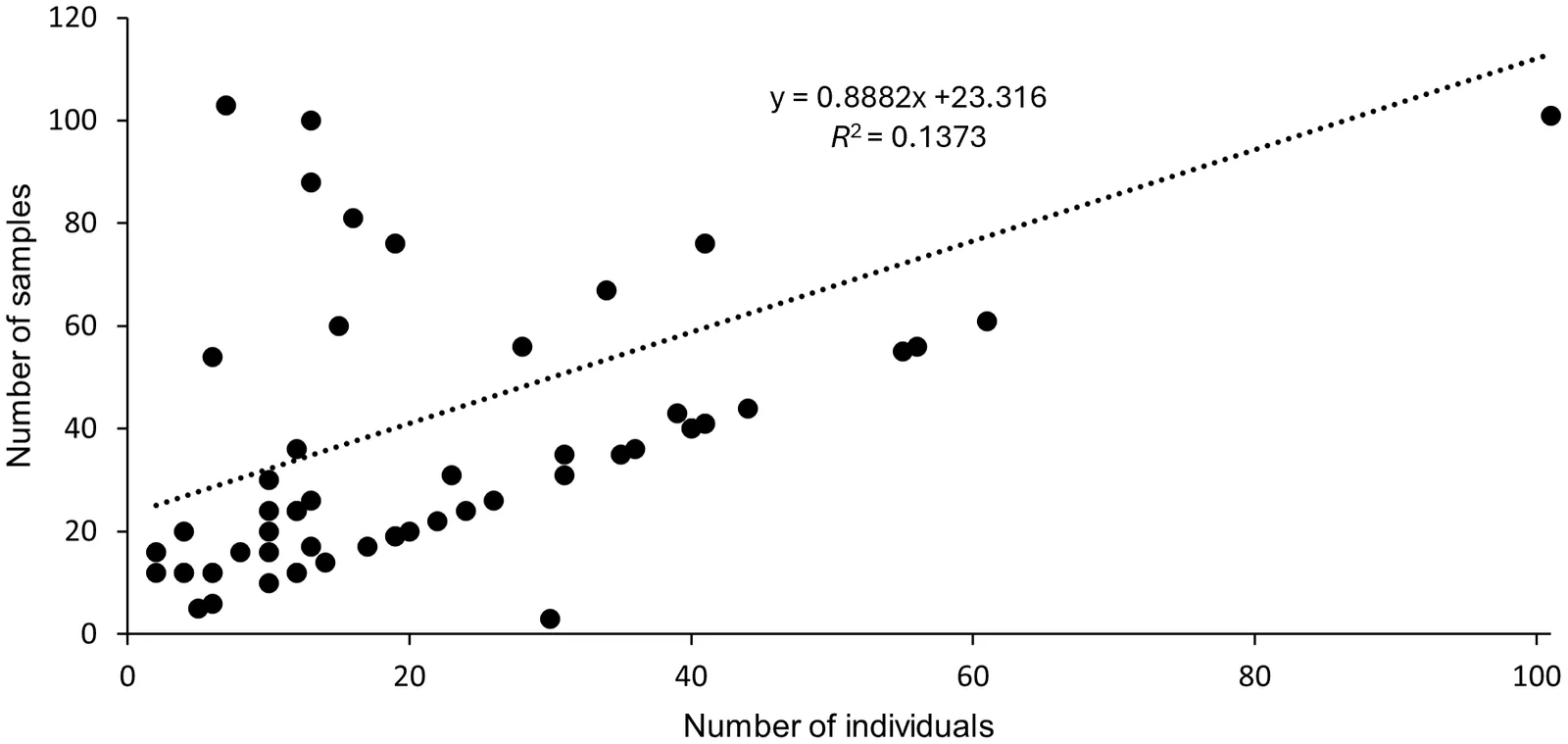
Scatter plot illustrating the relationship between the number of individuals and the number of samples in non-invasive mammalian metabolomics publications. Each point represents a single study unless sample number or individual number was ND (*n* = 59). The x-axis shows the number of individual animals, and the y-axis shows the total number of samples analysed, the dashed line represents the line of regression fit (y = 0.889x + 23.316; *R*^2^ = 0.137).

### Current trends and gaps in non-invasive biological sample types across taxonomic groups

Three mammalian papers utilised more than one sample type, bringing the total number of biological samples across all publications to 71. Sample types for Mammalia included liquid secretions (2/68(71); 2.9%), including both vaginal and anogenital gland, saliva (1/68(71); 1.5%), milk (1/68(71), and urine (18/68(71); 26.5%); solid or semi-solid material including faecal excreta (44/68(71); 64.7%) and hair (2/68(71); 2.9%); and volatile outputs including body odour (1/68(71); 1.5%) and breath (3/68(71); 4.4%). Sample type varied with taxonomic class (Fig 6).

**Fig 6:**
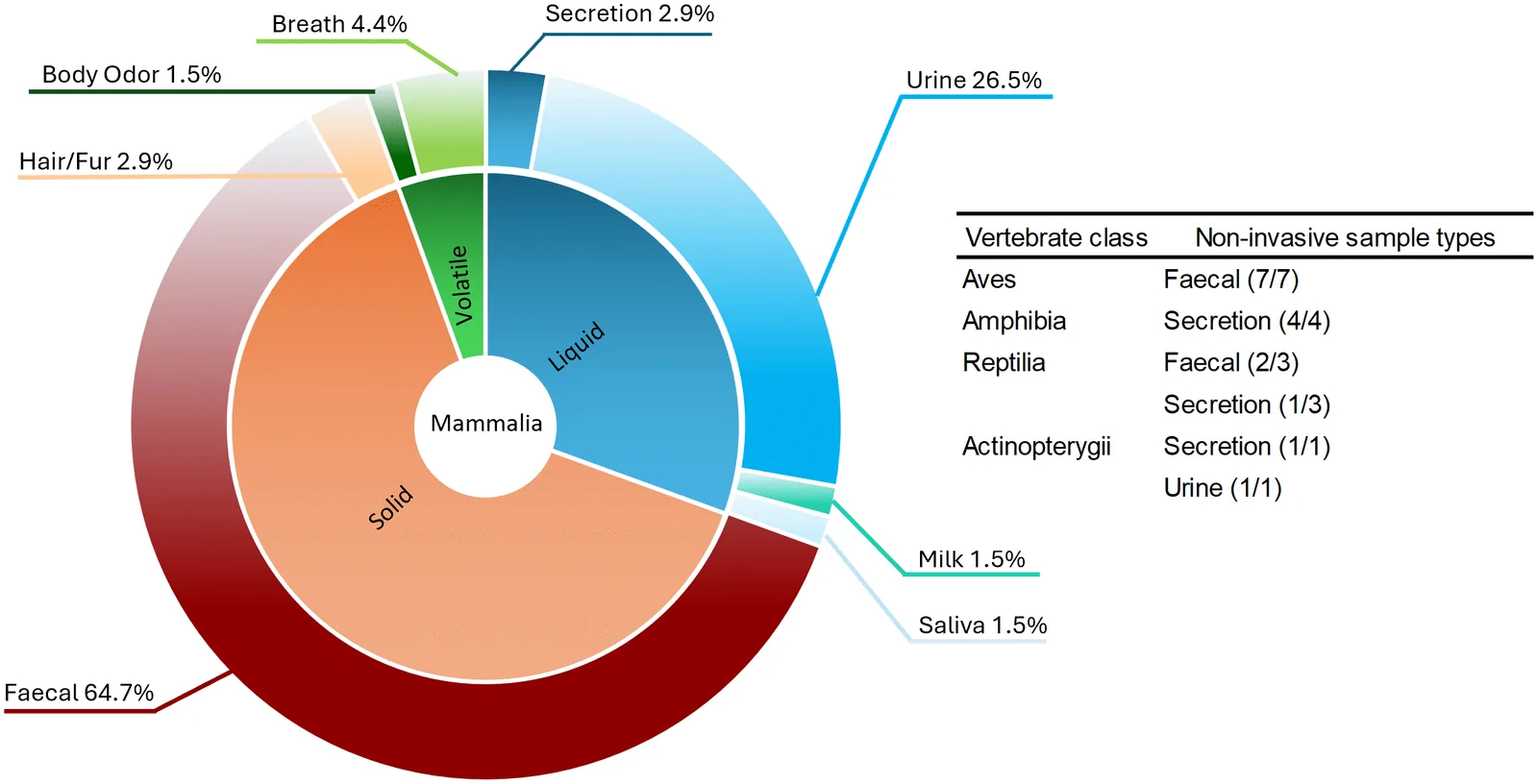
Distribution of non-invasive sample types used for vertebrate wildlife metabolomic studies included in this review. The pie chart illustrates the proportion of non-invasive sample types used in mammalian studies by state (*n* = 71 sample types in *n* = 68 publications), solid (orange; 67.6% (hair; 2.9% and faecal; 64.7%)), liquid (blue; 32.4% (secretion; 2.9%, urine; 26.5%, milk; 1.5% and saliva; 1.5%)) and volatile (green; 5.9% (breath; 4.4% and body odour 1.5%) The accompanying table provides the non-invasive sample types used for less represented taxa (Aves, Amphibia, Reptilia and Actinopterygii).

Faecal sampling was not only the most common sample type investigated in mammalian publications, but also the sample type utilised in all bird and one of two reptilian publications. This preference is mirrored throughout non-invasive wildlife research [19], reflecting not only its relative ease of collection compared with other sample types, but its versatility in pathogen detection, diet analysis, pollutant detection and reproductive (hormone-based) status assessment. In both bird and reptile species, faecal samples have been shown as a valuable sample type in microbiome studies, with samples containing more diverse microbial communities when compared with cloacal swab sampling, perhaps partially responsible for the selection of this sample type, with many publications opting to conduct multi-omics studies [61,98,100]. Wild bird ringing programs present an opportunity for faecal sample collection alongside ongoing research activities. As over 800,000 wild birds were caught and ringed in regions of the UK during 2024 (https://www.bto.org), perhaps physiological assessments recorded during ringing would benefit from more frequent coupling with metabolomic analysis, enhancing current research [94]. Further to this, the measurement of faecal glucocorticoid metabolites (i.e., cortisol or corticosterone) is widely used across a vast variety of vertebrate species as a tool to monitor stressful events, with over 1,300 published papers from 1996-November 2018 [126], many of which wildlife studies where metabolomics could provide deeper insight into faecal analyte composition.

Hair (or fur) was utilised in two mammal publications and is often used to investigate contaminant or drug residue in wildlife species due to it being comparatively an excellent sample for long term exposure; it also has the added benefit of being easily collected and stored. As metabolomics is being increasingly applied in environmental toxicological studies, these studies (both published in the last four years) may represent an emerging non-invasive sample type in wildlife metabolomics. Feathers have also been explored in wildlife studies outside metabolomics for the detection of contaminants such as heavy metals [127]; non-invasive markers of stress in poultry feather pulp have also been characterized recently, perhaps highlighting this as an underutilised sample in wildlife metabolomic studies, where methods have already been developed [128]. In addition, a recent publication has evaluated skin surface sampling methods for human metabolomic studies [129], skin scraping or taping can retrieve dead superficial skin cells, a sample type which has only been previously used in parasitic disease assessment in wildlife species [127], highlighting a further non-invasive sample which could perhaps be utilised in future research.

Urine excretion in mammals and secretion collection across other taxonomic groups was also common. Like faeces, urine is a widely accepted biological sample, already used to assess a diverse range of physiological parameters in wildlife species. Urine can often be collected non-invasively from the ground or foliage or minimally invasively via catheterization if an animal is anaesthetised for other reasons. Although urine can be collected from amphibians during handling, and has been used for other conservation physiology research, amphibian urine studies were not present in this review and could represent a potentially underutilised non-invasive sample type for amphibian metabolomics [130].

Metabolomic assessment of skin mucus or secretions has been used for both amphibian and reptile species included in this review [131]. Although the methodology used can vary, protocols often use a cotton dry tipped swab which is rolled over the ventral and dorsal surfaces of the skin or alternatively, placing the animal in sterile water for a standardised period. Skin mucus represents an important resource for non-invasive monitoring of health in amphibian populations, including chytridiomycosis, a disease caused by the fungal pathogen *Batrachochytrium dendrobatidis* and other emerging infectious diseases.

Despite milk metabolomics having broad application in both nutrition and reproductive success, conclusions beyond the recent characterisation of interspecies differences are lacking and require method consolidation and further sampling [68]. Although milk has emerged as an important biological sample in both dairy cattle and human metabolomics studies in recent years, there remains a clear call for standardisation of study design, despite focused research efforts within these fields [68,132], demonstrating parallels between human, domestic and wildlife species research gaps.

Blow and breath collection, although described for a range of species elsewhere, has seldom been used for metabolomic analysis and was present in only three marine mammal publications. Despite published methodology in 2014, low uptake in free ranging cetaceans could be attributed to complex collection apparatus and standardisation difficulties, perhaps leading researchers to prioritise more robust matrices, which provide a higher metabolite abundance [109].

### Current trends and gaps in metabolomics analytical techniques utilised

Choosing the optimal analytical technique for the metabolomic analysis of biological sample types can be challenging; no single approach is able to capture the whole metabolome in any given sample, due to the highly heterogeneous composition and broad range of metabolites present, each with differing physicochemical properties. The publications included in this review have used a variety of analytical techniques for metabolomics analyses. Current trends in metabolomics technologies for natural samples typically include LC-MS and GC-MS. The choice of these techniques is that MS, with prior chromatographic separation of metabolites, allows good selectivity and sensitivity for the identification and quantification of metabolites. LC-MS was the single technique used most frequently for metabolomic analysis in publications reviewed (47/83; 56%), likely due to the ability to detect and identify a diverse range of metabolites with the availability of both hydrophilic interaction chromatography (HILIC) providing separation of polar/hydrophilic compounds and reversed-phase methods for the separation of nonpolar/lipophilic compounds. GC-MS was the second most frequently used standalone technique (16/83; 19%), delivering improved metabolite separation of compound classes such as amino acids, fatty acids, and carbohydrates and benefitting from robust, standardized spectral libraries for metabolite identification.

Nuclear magnetic resonance (NMR) spectroscopy alone was utilised in only nine (11%) of the publications reviewed; other spectroscopy techniques such as Fourier transform infrared (FTIR) or Raman spectroscopy, allowing rapid and non-destructive information on compounds present in unprocessed samples or extracts, were not present [133]. It could be theorized that spectroscopic techniques such as NMR are underutilised in non-invasive wildlife metabolomic research because wildlife metabolomes are currently poorly characterised, leading researchers to prioritise maximising feature detection through increased sensitivity and breadth of recovery of LC-MS [134,135]. However, it could conversely be argued that spectroscopic techniques permit comparatively rapid and reproducible analysis with minimal sample preparation required and therefore could be currently underutilised [136–138].

Liquid chromatography ultraviolet visible spectroscopy (LC-UV-Vis) was used in three publications (4%), although this technique is considerably more economical than LC-MS it lacks sensitivity and specificity by comparison, particularly when considering complex biological sample matrixes.

Due to the analytical biases highlighted, combined approaches are often used in model animal studies to produce complementary results, increasing metabolite coverage in complex biological samples [139]. In addition, LC-MS suffers from challenges with respect to metabolite identification, due to the choice of mobile and stationary phased used in LC [140], and GC-MS is constrained by volatility and polarity of compounds and thus typically requires chemical derivatisation prior to analysis. Eight (10%) of the publications included in this review utilised more than one analytical technique for metabolomic analysis. Six studies utilised both GC and LC-MS techniques allowing both hydrophilic and hydrophobic small molecules to be analysed in a complementary fashion; two further studies combined the specificity and sensitivity of LC-MS with the highly reproducible and non-destructive NMR. The incorporation of multiple techniques analysis in future studies will allow for a more comprehensive analysis of metabolome compositions; however, the cost, both environmentally and financially of using multiple techniques should be taken into consideration.

Metabolite identification remains one of the most challenging steps in metabolomics studies, particularly in untargeted studies, with the rate of spectral data annotation across the majority of analytical platforms and matrixes remaining low (2–10% confidently annotated) [141]. To annotate metabolite features with identifiers, a data source is needed for comparison and at least two independent and orthogonal variables (e.g., retention index and mass spectrum) [142]. However, this often limits researchers to the identification of commercially available metabolites only, forcing researchers to sideline numerous potentially important metabolites. Scientists should be encouraged to publish their spectra in an open format to increase information available.

### Metabolomics with other technologies: Microbiome

Although a powerful tool, metabolomics alone of course cannot provide a whole view of the complex array of molecules and thus their biochemical interactions in any given biological sample [56]. Over the past decade, the importance of the microbiome in particular and its critical role in the physiology, health and nutrition of animal wildlife has been a topic of great interest, with the application of non-invasive sampling methods permitting the rapid emergence of the term ‘conservation metagenomics’ [143]. Almost 60% (48/83) of publications captured in this review have incorporated metagenomics, utilising phylogenetic profile analysis to provide insight into how the microbial communities of a given sample are influencing metabolites and thus metabolic pathways.

### Current trends and gaps in research areas

The publications included in this review were categorised into five broad research areas. (1) analytical techniques – metabolomic method development; (2) physiology and reproduction – characterisation of physiological metabolomic profiles including those associated with reproduction (for example sex, age or reproductive cycle stage); (3) diet and nutrition – studies related to diet and/or nutrition (for example wild *vs.* captive animal diet); (4) ecology and evolution – research linking metabolomics to geographical location or adaptation (for example high altitude adaptation) and (5) health and disease states – research examining metabolomic profiles in relation to the identification of healthy or disease states (for example stress response). These areas are briefly discussed below (Fig 7).

**Fig 7:**
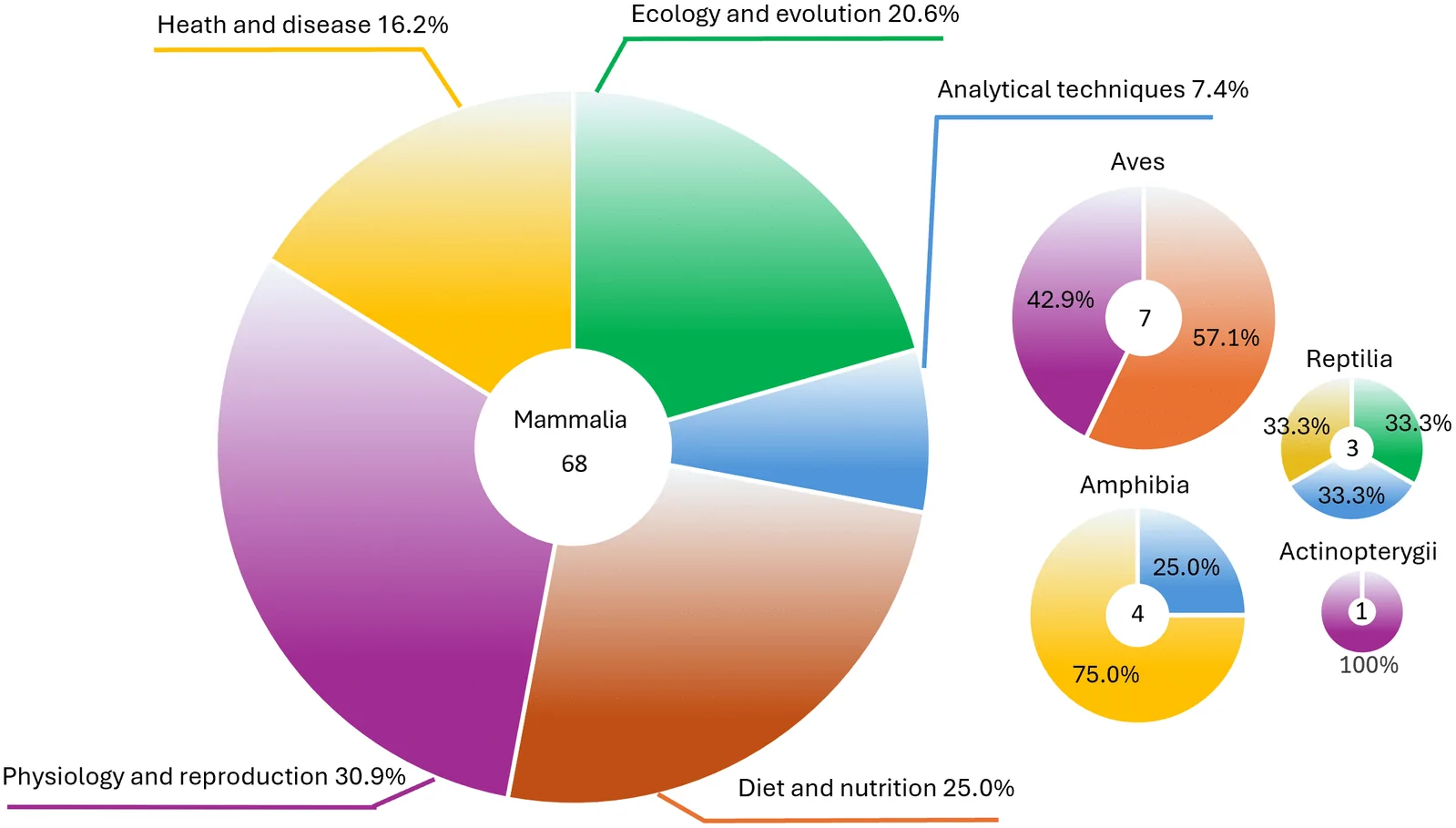
Pie charts illustrating the proportion of wildlife metabolomics publications by research area and taxonomic group. Mammalia (*n* = 68), health and disease (yellow, 16.2%); ecology and evolution (green, 20.6%), physiology and reproduction (pink, 30.9%) and diet and nutrition (brown, 25.0%) and analytical techniques (blue, 7.4%). Aves (*n* = 7), physiology and reproduction (pink, 42.9%) and diet and nutrition (brown, 57.1%). Amphibia (*n* = 4), health and disease (yellow, 75.0%) and analytical techniques (blue, 25.0%). Reptilia (*n* = 3), health and disease (yellow, 33.3%); ecology and evolution (green, 33.3%); analytical techniques (blue, 33.3%). Actinopterygii (*n* = 1), physiology and reproduction (pink, 100.0%).

The most popular area of research was physiology and reproduction (25/83; 30%), which is perhaps not surprising given the drive to conserve endangered species. This included 21 mammal publications, 10 of which focused on the giant panda [36,37,39,41,42,45–47,49,50]; the most studied species of all publications included in this review. The remaining publications were made up of three bird and one fish study. In both the giant panda and the finless porpoise (*Neophocaena asiaeorientalis*) metabolomic differences between reproductive stages and pregnancy were characterized. This research provides a metabolomics technique for the management of timed introductions for mating success and pregnancy detection in captivity and additionally offers valuable insights into global metabolic changes and thus physiological requirements during reproductive stages, allowing for closer examination of nutritional requirements during these states [84,85].

Giant panda research has expanded upon this foundation with the exploration of environment induced mate selection, stress-related metabolic pathways in relation to mating behaviour and incompatible pairing through the assessment of metabolomic changes to improve reproductive success rates [39,41,46]. Semiochemical communication was also explored in several other species, including maned wolf (*Chrysocyon brachyurus*) [60], house mouse (*Mus musculus*) [82] and great lakes char *(Salvelinus namaycush*) [101]. This investigation of novel, species-specific reproductive mechanisms utilising chemical cues is vital for the conservation of declining species, particularly for solitary animals that rely upon these mechanisms for reproductive success.

Several publications assessed other physiological characteristics such as sex or age. Both the urinary metabolome of captive giraffe (*Giraffa camelopardalis*) and the faecal metabolome of free-ranging giraffe have shown the metabolome to vary dependant with age, sex and pregnancy, with changes in diet causing clear variation between faecal metabolome profiles observed [66,67]. Similar studies have investigated these traits in the giant panda, cheetah (*Acinonyx jubatus*) [31], brown bat (*Eptesicus fuscus*) [65], meerkat (*Suricata suricatta*) [105], killer whale (*Orcinus orca*) [90], African penguin (*Spheniscus demersus*) [104] and great tit (*Parus major*) [94]. First insights into these species-specific characteristics provide specific findings that can aid population management – for example, facilitating the effective management of older individuals within captive populations. These data also enable researchers to develop future study designs with a foundation of baseline metabolomic knowledge for a given species (and sample type).

The second most prevalent research area was diet and nutrition (22/83; 27%), comprising of 17 mammal, four bird and one reptile study. Numerous studies sought comparisons of both the microbiome and metabolome of captive *vs*. free-ranging populations with examples including reindeer (*Rangifer tarandus*) [97], leopard (*Panthera pardus*) [92], monal (*Lophophorus lhuysii*) [75], rock ptarmigan (*Lagopus muta japonica*) [71] and pangolin species (*Manis pentadactyla* and *Manis javanica*) [80]; further studies focused on captive dietary adaptation in both the giant panda [38,43,44,51] and the finless porpoise [86]. Dietary supplementation was also considered in both Baird’s tapir (*Tapirus bairdii*) [106] and cheetah [30]. As captivity influences gut microbial composition and thus the faecal metabolome compared with free-roaming counterparts, characterisation of these variances is vital for the success of captive populations which could be translocated as part of holistic approaches to maintain genetically diverse populations *in situ*.

Numerous publications (18%; 15/83) focused on ecology and evolution, comprising of 14 mammal and one reptile study. Species-specific taxonomic discrimination of several mustelid species through metabolic profiles has been achieved, providing additional important non-invasive approaches for further study [64]. Environmental constraints have been shown to alter metabolomic profiles in wood rats (*Neotoma bryanti*) [89], rhesus macaques (*Macaca mulatta*) [78] and gorilla species (*Gorilla gorilla*) [69,70] in response to climate change, high-altitude and geographical isolation, respectively. The effect of environmental condition on the skin metabolome of adult spring peepers (*Pseudacris crucifer*) has also been investigated [96]. These studies reveal how species adjust their metabolism to environmental change and could help identify vulnerable populations or hidden nutritional limitations, informing targeted management actions which could include habitat protection or dietary supplementation.

Fifteen publications (15/83; 18%) focused on health and disease, comprising of 11 mammal, three amphibian and one reptilian study. The disease management practice of deworming in primate species has been shown to alter gut microbiome-metabolite interactions [98]. Hair metabolomic profiling in forest musk deer (*Moschus berezovskii*) has highlighted this sample type as a potential avenue for detecting metabolic alterations associated with disease [81]. These studies build upon baseline characterization, highlighting the capability of metabolomics as a tool for longitudinal wildlife health monitoring.

Several studies explored species-specific markers of stressful situations, rhesus macaque (*Macaca mulatta*) [76], bottlenose dolphin (*Tursiops truncatus*) [108], finless porpoise [87] and saltwater crocodile (*Crocodylus porosus*) [61]. In saltwater crocodiles, hormone profiling of faecal glucocorticoids has been combined with metabolomics and microbiome analysis in a multifaceted approach emphasizing the capability of -omics methodologies through the identification of both metabolome and microbiome perturbations, which were undetected in faecal corticosterone levels.

Multi-omics has also been utilised in the assessment of fungal infection in amphibians; publications in experimental and field settings have been shown to robustly predict *Batrachochytrium dendrobatidis* disease dynamics with novel candidate biomarkers of infection identified in the common midwife toad (*Alytes obstetricans*) [54]. Population-level variation in skin metabolite profiles have highlighted differences that may affect disease susceptibility with analysis in threatened species such as the rocket frog (*Silverstoneia flotator*) [102] demonstrating the potential of these approaches as predictive resources for amphibian conservation.

Although some publications did include metabolome comparisons between biological sample types, this review identified only six publications (6/83; 7%), which focused on the development and description of analytical technique as the primary objective of the research. This number is surprisingly few, given the importance placed on consistency of both pre analytical and extraction techniques within both human and model animal metabolomics publications and reviews [144]. Breath metabolite analysis, a non-invasive method developed and optimised for monitoring cetacean health, both in managed populations and in the wild was noted in bottlenose dolphins [109]. Additionally, extraction solvents were explored in three giant panda publications (fur and faecal) [35,40,48] where multiple extraction methods were investigated and evaluated for their reproducibility, metabolite coverage, and extraction efficiency. There were no examples found where the impact of storage conditions, freeze thawing, voiding to collection latency (faecal or urine) or environment conditions had been directly assessed, although these are likely to impact metabolomic data, particularly when considering the variability and challenges of field sample collection [144,145]. Considering the plethora of vertebrate wildlife species, of which many appear singularly in this review, and the known complexity of both non-invasive sample matrices and the heterogenous nature of the metabolome, species-specific metabolite extraction optimisation studies could maximise breadth and consistency of feature recovery, allowing standardisation of such methods, enabling future comparison and thus improving research impact.

## Discussion

The aim of this review was to gain vital insight into how metabolomics may be contributing to the health, well-being and ultimately the conservation of wild vertebrate animal species, subsequently identifying and reviewing 83 peer-reviewed publications in relation to this subject area. Metabolomics is well placed to advance vertebrate wildlife research by enabling evidence-based conservation action, with numerous non-invasive biological sample types allowing the assessment of the metabolome without compromising the welfare of individuals in vulnerable populations. However, realising its full potential will require further development. As this review highlights, there are gaps in wildlife metabolomics research including the consideration of metabolite extraction optimisation, storage conditions, the use of integrated omics platforms and consideration of statistical power, particularly in respect to intra-individual variation and the characterisation of species-specific metabolomes.

The number of subjects available will understandably always be a major limitation of wildlife research, particularly those of conservation interest, when compared with human, livestock and laboratory animal models. It may be virtually impossible to obtain large discovery and validation cohorts of samples ordinarily required to facilitate true ‘biomarker’ discoveries in wildlife species. In order to combat this, alternative approaches are required, which could include increasing confidence in the biological relevance of markers through multi-matrix validation, that is, showing a metabolic signature associated with a particular physiology consistently across multiple biological sample types. Several studies included in this review compared multiple biological sample types and although blood sampling was not within this scope of this review, the inclusion of circulating metabolites, where feasible, would allow for more stringent physiological validation of identified markers.

The giant panda is by far the most studied species included in this review, with multiple non-invasive sample types explored and progress toward a species-specific metabolomic database underway [37]. This sets a strong foundation and touch stone for which future metabolomic research can reference and build upon in this species. This work promotes data traceability and reproducibility, a concept that has long been accepted for both human (database creation in 2007 human metabolome database (HMDB); https://www.hmdb.ca) and livestock (2017 LMDB; https://lmdb.ca) metabolomics research.

Regardless of research area, many publications combined metabolomics and metagenomics (microbiome analysis) methodologies, highlighting an emerging trend in wildlife research. Given the importance of the microbiome in both human and animal clinical research settings, wildlife studies would likely benefit from alignment with already well represented model animal metabolome research experimental design [146]. This review, although limited by search term use in its breadth of article recovery, gives the first broad insight into non-invasive vertebrate wildlife metabolomics.

### Strengths and limitations

The strengths of this review include the use of multiple and considered databases and the adherence to a predesigned protocol, meaning the scope of the search was appropriately focused, effectively balancing resource feasibility and comprehensiveness of the review.

This review was not designed to be exhaustive, but to broadly map existing literature characteristics and highlight potential gaps in how metabolomics is being applied non-invasively in wild vertebrate animal species. As such, the scope of this review was broadly inclusive of all vertebrate species, and it is recognised that the absence of focused taxonomic, family or order searches, particularly in non-mammalian species, represents significant limitation and is likely to have impeded retrieval of some sources, such limitations are further described in S4 Appendix.

Furthermore, we did not explore an assessment of quality beyond reference to sample and subject number as part of this scoping review; the assessment of mass spectrometry technique employed, targeted *vs.* untargeted methodology, metabolite quantification or identification and statistical analysis would provide further insight and could be considered by future reviewers.

## Conclusion

It is hoped that this review will stimulate future investigation, building upon the general trends and gaps highlighted and give further insight into details such as metabolite identification, quantification and the breadth of metabolite coverage within non-invasive vertebrate wildlife metabolomics research. It has been highlighted that the future of metabolomics relies on researchers learning to effectively synthesize existing knowledge to guide hypothesis-driven research [147]. Perhaps this review can serve as a call to action, to ensure the current learnings and potential of this rapidly developing field is harnessed successfully in the fight to conserve wildlife species.

## Supporting information

S1 Checklist Preferred Reporting Items for Systematic reviews and Meta-Analyses extension for Scoping Reviews (PRISMA-ScR) checklist. (PDF)

S2 File Search strategy. Sample search strategy for Scopus.
(DOCX)

S3 File Species table. All species included in the review.
(DOCX)

S4 Appendix Study limitations. (DOCX)
