# Supplementary material for: Metabolomics in the wild: A scoping review of non-invasive methods for understanding vertebrate wildlife health and success
Source: PLoS One. 2026 Oct 26;21(10):e0359720. doi: 10.1371/journal.pone.0359720 (PMC13704869; doi:10.1371/journal.pone.0359720)
Supplement: Supplementary file 2 [file pone.0359720.s002.docx]

Search strategy Database 1: Scopus

Initial searches were performed via the advanced search function in Scopus:

Initial search was restricted to title, abstract and key words and contained the following components:

Initial Scopus advanced query on 02.07.25 and retrieved 73,117 documents

(a) metabolomics as the field of research
(b) Wildlife as the research subject

Boolean string:

*TITLE-ABS-KEY (metabolom* OR metabonom* OR ( metab* AND fingerprint* ))*

*AND (animal* OR wildlife OR wild OR exotic OR zoo* )*

*AND (PUBYEAR > 2010 AND PUBYEAR < 2026)*

Following this, five documents were selected from the search to cover the breadth of the acceptance criteria (Table 1 below) to integrate the search strategy following refinement with identified keywords and index terms for Scopus (and each included database in the same way) to increase search efficiency and filter irrelevant results. To enhance the sensitivity and specificity of the search the addition of terms like ‘captive’ allowed further documents that reference common family or order animal names instead of broad taxonomic categories without the introduction of specific taxa bias to the search (as not all species can be included within the search terms).

**Table 1:** Appendix I Search strategy Database 1: Documents selected to interrogate search

| **Title** | **Citation** | **Year pub.** | **DOI** |
| --- | --- | --- | --- |
| First Insights into the fecal Metabolome of Healthy, Free-Roaming Giraffes (Giraffa camelopardalis): An Untargeted GCxGC/TOF-MS Metabolomics Study | *Grobbelaar A, Osthoff G, du Preez I, Deacon F. First Insights into the Fecal Metabolome of Healthy, Free-Roaming Giraffes (Giraffa camelopardalis): An Untargeted GCxGC/TOF-MS Metabolomics Study. Metabolites. 2024 Oct 28;14(11):586. doi: 10.3390/metabo14110586. PMID: 39590822; PMCID: PMC11596133.* | 2024 | DOI:10.3390/metabo14110586 |
| Utilizing NMR fecal metabolomics as a novel technique for detecting the physiological effects of food shortages in waterfowl | *Murray BA, Machin KL. Utilizing NMR fecal metabolomics as a novel technique for detecting the physiological effects of food shortages in waterfowl. Front Physiol. 2024 Jan 10;14:1229152. doi: 10.3389/fphys.2023.1229152. PMID: 38269059; PMCID: PMC10806059.* | 2024 | DOI:10.3389/fphys.2023.1229152 |
| Neotropical mustelids: fecal metabolome diversity and its potential for taxonomic discrimination | *Almeida LR, Alves MA, Mastella AMO, Garrett R, Pereira MJR. Neotropical mustelids: fecal metabolome diversity and its potential for taxonomic discrimination. Integr Zool. 2023 May;18(3):518-529. doi: 10.1111/1749-4877.12645. Epub 2022 Apr 15. PMID: 35275446.* | 2022 | DOI: 10.1111/1749-4877.12645 |
| Variation in Metabolite Profiles of Amphibian Skin Bacterial Communities Across Elevations in the Neotropics | *Medina D, Hughey MC, Becker MH, Walke JB, Umile TP, Burzynski EA, Iannetta A, Minbiole KPC, Belden LK. Variation in Metabolite Profiles of Amphibian Skin Bacterial Communities Across Elevations in the Neotropics. Microb Ecol. 2017 Jul;74(1):227-238. doi: 10.1007/s00248-017-0933-y. Epub 2017 Jan 19. PMID: 28105509.* | 2017 | 10.1007/s00248-017-0933-y |
| Metabolite Content Profiling of Bottlenose Dolphin Exhaled Breath | *Anal. Chem. 2014, 86, 21, 10616–10624* | 2014 | DOI: 10.1021/ac5024217 |

Improved advanced Scopus query on 31.01.2026

(a) Metabolomics as the field of research
(b) Wildlife as the research subject
(c) Non-invasive sampling specification
(d) The time frame
(e) Addition of a secondary search specifically including mammal order and family names, the justification of which is below

A secondary search in Scopus was conducted using all extant mammal orders and families (Mammal Diversity Database, 2023) to maximise retrieval of relevant literature. Other vertebrate classes were not included due to resource constraints and lower prevalence of wildlife indexed studies. Order names were wildcarded where appropriate and searched in title/abstract fields to identify records for mammalia where taxonomic names have been used instead of topic-based terms. Results were then merged with the primary search and deduplicated before screening.

Primary Boolean string (199 Articles):

(TITLE-ABS-KEY (metabolom* OR metabonom* OR "metab* fingerprint*" OR "metabolite profile*" OR "metabolite profiling" OR "metabolite analysis" OR "metabolic profile*")

AND (TITLE-ABS-KEY ("wildlife" OR "wild animal*" OR "zoo*" OR "exotic animal*" OR "free-ranging" OR "free ranging" OR "free-living" OR "free living" OR "non-domestic" OR "non domestic" OR "captiv*" OR "wild mammal*" OR "wild bird*" OR "wild amphibian*" OR "wild fish*" OR "wild reptile*" OR "wild vertebrate*"))

AND (TITLE-ABS-KEY ("non-invasive" OR "noninvasive" OR feces OR faeces OR faecal OR fecal OR scat* OR droppings OR urine OR urinary OR feather* OR hair* OR saliva OR skin OR mucus OR "mucus swab*" OR "cloacal swab*" OR scale* OR breath OR blow OR respiratory OR egg*))

(PUBYEAR > 2010 AND PUBYEAR < 2026) AND NOT (TITLE-ABS-KEY (human*)))

Secondary Boolean string (122 articles):

(TITLE-ABS-KEY (metabolom* OR metabonom* OR "metab* fingerprint*" OR "metabolite profile*" OR "metabolite profiling" OR "metabolite analysis" OR "metabolic profile*")

AND (TITLE-ABS-KEY ("wildlife" OR "wild animal*" OR "zoo*" OR "exotic animal*" OR "free-ranging" OR "free ranging" OR "free-living" OR "free living" OR "non-domestic" OR "non domestic" OR "captiv*" OR "wild mammal*" OR "wild bird*" OR "wild amphibian*" OR "wild fish*" OR "wild reptile*" OR "wild vertebrate*" OR Monotremata OR Didelphimorphia OR Paucituberculata OR Microbiotheria OR Dasyuromorphia OR Peramelemorphia OR Notoryctemorphia OR Diprotodontia OR Afrosoricida OR Macroscelidea OR Tubulidentata OR Hyracoidea OR Proboscidea OR Sirenia OR Cingulata OR Pilosa OR Primates OR Rodentia OR Lagomorpha OR Eulipotyphla OR Chiroptera OR Carnivora OR Perissodactyla OR Artiodactyla OR Ornithorhynchid* OR Tachyglossid* OR Dasyurid* OR Myrmecobiid* OR Didelphid* OR Acrobatid* OR Burramyid* OR Macropodid* OR Petaurid* OR Phalangerid* OR Phascolarctid* OR Potoroid* OR Pseudocheirid* OR Tarsipedid* OR Vombatid* OR Notoryctid* OR Peramelid* OR Thylacomyid* OR Caenolestid* OR Microbiotheriid* OR Chrysochlorid* OR Tenrecid* OR Macroscelid* OR Orycteropodid* OR Elephantid* OR Dugongid* OR Trichechid* OR Procaviid* OR Chlamyphorid* OR Dasypodid* OR Bradypodid* OR Choloepodid* OR Cyclopedid* OR Myrmecophagid* OR Erinaceid* OR Solenodontid* OR Soricid* OR Talpid* OR Emballonurid* OR Furipterid* OR Hipposiderid* OR Megadermatid* OR Miniopterid* OR Molossid* OR Mormoopid* OR Mystacinid* OR Natalid* OR Noctilionid* OR Nycterid* OR Phyllostomid* OR Pteropodid* OR Rhinolophid* OR Rhinopomatid* OR Thyropterid* OR Vespertilionid* OR Ailurid* OR Canid* OR Euplerid* OR Felid* OR Herpestid* OR Hyaenid* OR Mephitid* OR Mustelid* OR Odobenid* OR Otariid* OR Phocid* OR Procyonid* OR Ursid* OR Viverrid* OR Equid* OR Rhinocerotid* OR Tapirid* OR Antilocaprid* OR Balaenid* OR Balaenopterid* OR Bovid* OR Camelid* OR Cervid* OR Delphinid* OR Eschrichtiid* OR Giraffid* OR Hippopotamid* OR Iniid* OR Kogiid* OR Monodontid* OR Moschid* OR Neobalaenid* OR Phocoenid* OR Physeterid* OR Platanistid* OR Pontoporiid* OR Suid* OR Tayassuid* OR Tragulid* OR Ziphiid* OR Manid* OR Aotid* OR Atelid* OR Callitrichid* OR Cebid* OR Cercopithecid* OR Cheirogaleid* OR Daubentoniid* OR Galagid* OR Hominid* OR Hylobatid* OR Indriid* OR Lemurid* OR Lepilemurid* OR Lorisid* OR Pitheciid* OR Tarsiid* OR Leporid* OR Ochotonid*))

AND (TITLE-ABS-KEY ("non-invasive" OR "noninvasive" OR feces OR faeces OR faecal OR fecal OR scat* OR droppings OR urine OR urinary OR feather* OR hair* OR saliva OR skin OR mucus OR "mucus swab*" OR "cloacal swab*" OR scale* OR breath OR blow OR respiratory OR egg*))

(PUBYEAR > 2010 AND PUBYEAR < 2026) AND NOT (TITLE-ABS-KEY (human*)
