# Supplementary material for: Metabolomics in the wild: A scoping review of non-invasive methods for understanding vertebrate wildlife health and success
Source: PLoS One. 2026 Oct 26;21(10):e0359720. doi: 10.1371/journal.pone.0359720 (PMC13704869; doi:10.1371/journal.pone.0359720)
Supplement: Supplementary file 3 [file pone.0359720.s003.docx]

**All species included in this review and frequency of occurrence** **with IUCN Red List category**

| **Vertebrate class** | **Common name** | **Scientific name** | **Status^a^** | **Publications** | |
| --- | --- | --- | --- | --- | --- |
| Mammalia |  |  |  | |  |
| Primates | Black howler monkey | *Alouatta pigra* | EN | | 1 |
|  | Black-and-white ruffed lemur | *Varecia variegata* | CR | | 1 |
|  | Chimpanzee | *Pan troglodytes* | EN | | 1 |
|  | Coquerel's sifaka | *Propithecus coquereli* | CR | | 1 |
|  | Diademed sifaka | *Propithecus diadema* | CR | | 1 |
|  | Eastern woolly lemur | *Avahi laniger* | VU | | 1 |
|  | Golden snub-nosed monkey | *Rhinopithecus roxellana* | EN | | 2 |
|  | Gray snub-nosed monkey | *Rhinopithecus brelichi* | CR | | 1 |
|  | Guinan weeper capuchin | *Cebus olivaceus* | LC | | 1 |
|  | Indri | *Indri indri* | CR | | 1 |
|  | Lar gibbon | *Hylobates lar* | EN | | 1 |
|  | Mandrill | *Mandrillus sphinx* | VU | | 1 |
|  | Red ruffed lemur | *Varecia variegata rubra* | CR | | 1 |
|  | Rhesus macaque | *Macaca mulatta* | LC | | 3 |
|  | Weasel sportive lemur | *Lepilemur mustelinus* | VU | | 1 |
|  | Weddell's saddle-back tamarin | *Leontocebus weddelli* | LC | | 1 |
|  | Western lowland gorilla | *Gorilla gorilla gorilla* | CR | | 3 |
|  | Mountain gorilla | *Gorilla* *beringei beringei* | EN | | 1 |
|  |  |  | Total | | 18 |
| Carnivora | Arabian wolf | *Canis lupus* | LC | | 1 |
|  | African wild dog | *Lycaon pictus* | EN | | 1 |
|  | Black bear | *Ursus thibetanus* | VU | | 2 |
|  | Lion | *Panthera leo* | VU | | 1 |
|  | Lesser grison | *Galictis cuja* | LC | | 1 |
|  | Meerkat | *Suricata suricatta* | LC | | 1 |
|  | Cheetah | *Acinonyx jubatus* | VU | | 5 |
|  | Giant otter | *Pteronura brasiliensis* | EN | | 1 |
|  | Neotropical otter | *Lontra longicaudis* | NT | | 1 |
|  | Giant panda | *Ailuropoda melanoleuca* | VU | | 17 |
|  | Brown bear | *Ursus arctos* | LC | | 2 |
|  | Greater grison | *Galictis vittata* | LC | | 1 |
|  | Hyena | *Hyaena hyaena* | NT | | 1 |
|  | Jungle cat | *Felis chaus* | LC | | 1 |
|  | Maned wolf | *Chrysocyon brachyurus* | NT | | 1 |
|  | Leopard | *Panthera pardus* | VU | | 1 |
|  | Elephant seal | *Mirounga leonina* | LC | | 1 |
|  | Red panda | *Ailurus fulgens* | EN | | 1 |
|  | Sand cat | *Felis margarita* | LC | | 1 |
|  | South American coati | *Nasua nasua* | LC | | 1 |
|  | Tayra | *Eira barbara* | LC | | 1 |
|  | Tiger | *Panthera tigris* | EN | | 2 |
|  | Weddell seal | *Leptonychotes weddelli* | LC | | 1 |
|  |  |  | Total | | 23 |
| Proboscidea | African elephant | *Loxodonta africana* | EN | | 1 |
|  | Asian elephant | *Elephas maximus* | EN | | 1 |
|  |  |  | Total | | 2 |
| Rodentia | Bryant's wood rat | *Neotoma bryanti* | LC | | 1 |
|  | Desert wood rat | *Neotoma lepida* | LC | | 1 |
|  | House mouse | *Mus musculus* | LC | | 2 |
|  |  |  | Total | | 3 |
| Perissodactyla | Black rhinoceros | *Diceros bicornis* | CR | | 1 |
|  | Baird's tapir | *Tapirus bairdii* | EN | | 1 |
|  | Greater one-horned rhinoceros | *Rhinoceros unicornis* | VU | | 1 |
|  | Plains zebra | *Equus burchelli* | NT | | 1 |
|  | Sumatran rhinoceros | *Dicerorhinus sumatrensis* | CR | | 1 |
|  | White rhinoceros | *Ceratotherium simum* | NT | | 2 |
|  |  |  | Total | | 6 |
| Diprotodontia | Big brown bat | *Eptesicus fuscus* | *LC* | | 1 |
|  | Red kangaroo | *Osphranter rufus* | *LC* | | 1 |
|  | Seba's short tailed bat | *Carollia perspicillata* | *LC* | | 1 |
|  |  |  | Total | | 3 |
| Pholidota | Chinese pangolin | *Manis pentadactyla* | CE | | 1 |
|  | Sunda pangolin | *Manis javanica* | CE | | 1 |
|  |  |  | Total | | 2 |
| Cetartiodactyla | Red deer | *Cervus elaphus* | LC | | 1 |
|  | White lipped deer | *Cervus albirostris* | VU | | 1 |
|  | Blesbok | *Damaliscus pygargus* | LC | | 1 |
|  | Alpine ibex | *Capra ibex* | LC | | 1 |
|  | Reindeer | *Rangifer tarandus* | VU | | 1 |
|  | Giraffe | *Giraffa camelopardalis* | VU | | 3 |
|  | Forest musk deer | *Moschus berezovskii* | EN | | 1 |
|  | Finless porpoise | *Neophocaena asiaeorientalis* | EN | | 5 |
|  | Killer whale | *Orcinus orca* | DD | | 1 |
|  | Common bottlenose dolphin | *Tursiops truncatus* | LC | | 1 |
|  |  |  | Total | | 10 |
|  |  | Mammal | Total | | 67 |
| Aves | African penguin | *Spheniscus demersus* | CR | | 2 |
|  | Alexandrine parakeet | *Palaeornis eupatria* | LC | | 1 |
|  | Great tit | *Parus major* | LC | | 1 |
|  | Mallard duck | *Anas platyrhynchos* | LC | | 1 |
|  | Chinese monal | *Lophophorus lhuysii* | VU | | 1 |
|  | Rock ptarmigan | *Lagopus muta* | LC | | 1 |
|  |  |  | Total | | 6 |
| Reptilia | Frog-eyed gecko | *Teratoscincus roborowskii* | LC | | 1 |
|  | Spring peepers | *Pseudacris crucifer* | LC | | 1 |
|  | Saltwater crocodile | *Crocodylus porosus* | LC | | 1 |
|  |  |  | Total | | 3 |
| Amphibia | African foam-nest tree frog | *Chiromantis rufescens* | LC | | 1 |
|  | Buea screeching frog | *Arthroleptis variabilis* | LC | | 1 |
|  | Camp Kivu reed frog | *Hyperolius kuligae* | LC | | 1 |
|  | Common midwife toad | *Alytes obstetricans* | LC | | 1 |
|  | Dwarf toad | *Didynamipus sjostedti* | VU | | 1 |
|  | Eastern hellbenders | *Cryptobranchus alleganiensis* | VU | | 1 |
|  | Forest screeching frog | *Arthroleptis sylvaticus* | LC | | 1 |
|  | Foulassi banana frog | *Afrixalus paradorsalis* | LC | | 1 |
|  | Fraser's clawed frog | *Xenopus fraseri* | DD | | 1 |
|  | Golden puddle frog | *Phrynobatrachus auritus* | LC | | 1 |
|  | Modest tree frog | *Leptopelis modestus* | LC | | 1 |
|  | Ocellated spiny reed frog | *Hyperolius ocellatus* | LC | | 1 |
|  | Boulenger's striped frog | *Phlyctimantis boulengeri* | LC | | 1 |
|  | Rainforest rocket frog | *Silverstoneia flotator* | LC | | 1 |
|  | West African screeching frog | *Arthroleptis poecilonotus* | LC | | 1 |
|  | White-lipped frog | *Hylarana albolabris* | LC | | 1 |
|  |  |  | Total | | 16 |
| Actinopterygii | Great lakes char | *Salvelinus namaycush* | LC | | 1 |
|  |  |  | Total | | 1 |
