# Supplementary material for: Metabolomics in the wild: A scoping review of non-invasive methods for understanding vertebrate wildlife health and success
Source: PLoS One. 2026 Oct 26;21(10):e0359720. doi: 10.1371/journal.pone.0359720 (PMC13704869; doi:10.1371/journal.pone.0359720)
Supplement: Supplementary file 4 [file pone.0359720.s004.docx]

Limitations of this review

There are several limitations which should be considered when interpreting the findings of this review.

This review was not designed to be exhaustive, but to broadly map existing literature characteristics and highlight potential gaps in how metabolomics is being applied non-invasively in wild vertebrate animal species. As such, the scope of this review was broadly inclusive of all vertebrate species, and it is recognised that the absence of focused individual taxonomic searches may have impeded retrieval of some sources. However, data curation for this review was completed as part of an MPhil degree and thus limited in resource; furthermore, as not all publications necessarily use species name within keywords, was challenging to tackle this limitation fully. It was recognised that the identification of wildlife studies was limited by the selectivity of ‘wildlife’ as this is often implicit and not indexable, to address this, a secondary sensitivity search in Scopus was conducted using all extant mammal orders and families (Mammal Diversity Database, 2023) to maximise retrieval of relevant literature. Other vertebrate classes were not included due to resource constraints, complexity of taxonomy introducing bias and overall lower prevalence of wildlife indexed studies in these taxonomic groups.

Similarly, articles indexed with ‘human*’ were excluded from the search strategy as preliminary searches returned a high number of articles in relation to human comparisons or findings in relation to human health, which is outside the scope of this review. It is acknowledged that the exclusion of this term may exclude some non-human primate studies which are unrelated to human health; however, this decision was made to ensure the specificity of the search remained aligned with wildlife, and by conducting the secondary search on mammalian orders and families as described above, these should have been identified and included, where relevant.

Initial searches indicated that some relevant articles referenced ‘metabolite(s)’ as an indexable term without explicitly using metabolomics related terminology; however, this term was deliberately excluded from the search strategy as its inclusion yielded a high volume of out-of-scope articles, particularly studies with a focus on faecal glucocorticoids among similar hormone metabolite measurements and other immunoassays studies which did not meet the metabolomics definition applied and have been reviewed extensively elsewhere.
